# Evolution of ischemic stroke drug clinical trials in mainland China from 2005 to 2021

**DOI:** 10.1111/cns.13867

**Published:** 2022-06-01

**Authors:** Chunrui Bo, Tianqi Wang, Chengbei Hou, Jinming Han, Lixia Chen, Huixue Zhang, Lihua Wang, Hongyan Li

**Affiliations:** ^1^ Department of Neurology, XuanWu Hospital Capital Medical University Beijing China; ^2^ Department of Neurology, The Second Affiliated Hospital Harbin Medical University Beijing China; ^3^ Center for Evidence‐Based Medicine, Xuanwu Hospital Capital Medical University Beijing China; ^4^ Department of Neurology, Department of General Surgery, China National Clinical Research Center for Geriatric Diseases, XuanWu Hospital Capital Medical University Beijing China

**Keywords:** drug clinical trials, ischemic stroke, mainland China

## Abstract

**Background:**

To assess the temporal changes in the characteristics of ischemic stroke drug clinical trials conducted in mainland China in 2005–2021.

**Methods:**

A statistical analysis of registered clinical trials on ischemic stroke was performed using the platform of the Center for Drug Evaluation of China National Medical Products Administration, the Chinese Clinical Trial Registry, and ClinicalTrials.gov websites.

**Results:**

From January 1, 2005 to August 1, 2021, a total of 384 registered drug clinical trials on ischemic stroke were identified in mainland China. Over time, the number of trials gradually increased each year, with a significant growth in 2014, from 16 in 2013 to 42 in 2014. Phase IV trials (31.8%) accounted for the majority, followed by phase II (16.4%), phase I (10.9%), and phase III (8.6%). In terms of sponsorship, the proportion of investigator‐initiated trials (IITs) (60.7%) was higher than industry‐sponsored trials (ISTs) (39.3%). Additionally, trials involving traditional Chinese medicines (TCMs) (36.2%) accounted for the largest proportion, followed by trials involving antithrombotic therapy (19.5%) and cerebral protection agents (16.7%). Furthermore, over the past 17 years, the number of leading drug clinical trial units for ischemic stroke in mainland China has continuously increased. The leading principal units from Beijing, Shanghai, Guangdong, Jiangsu, and Liaoning accounted for the majority of the trials (67.4%).

**Conclusion:**

In the past 17 years, great progress has been made in the research and development (R&D) of drugs and clinical trials for ischemic stroke in mainland China. The most extensive progress was observed in TCMs, antithrombotic therapy, and cerebral protection agents. More clinical trials are needed to confirm whether the newly developed drugs can improve the clinical efficacy of ischemic stroke. Simultaneously, more pharmaceutical R&D efforts of innovative drugs are warranted.

## Graphical abstract

In the past 17 years, great progress has been made in the research and development of drugs and clinical trials for ischemic stroke in mainland China. Phase IV trials showed the greatest growth, with an average annual growth rate of 27.8%. The most extensive progress was observed in TCMs, antithrombotic therapy, and cerebral protection agents. This is the first study to depict the evolution of acute ischemic stroke drug clinical trials in mainland China in 2005–2021.

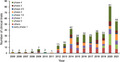

## 1 INTRODUCTION

Ischemic stroke is considered as the most common type of stroke, accounting for 69.6%–70.8% of stroke cases in China. The time division of the acute phase is not uniform. Generally, it refers to within 2 weeks after the onset of the disease, within 1 week of the mild type, and within 1 month of the severe type.^1^, ^2^ The case fatality rate of hospitalized patients with acute ischemic stroke (AIS) within 1 month after onset is about 2.3%–3.2%, and the case fatality rate at 3 months is 9%–9.0.6%, and the prevalence of disability/fatality is 34.5%–37.1%.^3^, ^4^

Prompt reperfusion is considered as the most effective treatment for AIS patients. Recent studies have revealed the benefit of extending the timing of reperfusion in carefully screened patients.^5^, ^6^ However, mechanical thrombectomy and intravenous thrombolysis may result in reperfusion injury after recanalization. Although basic and translational experiments have generated promising results, several neuroprotective agents do not improve the outcomes of clinical trials. Therefore, the government encouraged more drug research and development (R&D) for the treatment of ischemic stroke, which is very important to increase the efficacy of new drugs and to improve the clinical outcomes of ischemic stroke patients. Since 2015, the Chinese government has established regulatory policies that promote new drug development and clinical trials.^7^, ^8^, ^9^ A new R&D landscape for ischemic stroke drugs has then emerged, yet an overview of the analysis of drug clinical trials for ischemic stroke have not been performed to date.

Here, a summary was conducted to assess the evolution of ischemic stroke drug clinical trials in mainland China during the period of 2005–2021. Our work may provide clinical trial units with self‐assessment of research competitiveness and hospital development strategies. It would also provide insights and supportive findings for investigators, policymakers, pharmaceutical enterprises, and other stakeholders.

## 2 METHODS

### 2.1 Data sources

The data of registered clinical trials for ischemic stroke in mainland China were acquired through three websites: Center for Drug Evaluation (CDE) of China National Medical Products Administration (NMPA) website (http://www.cde.org.cn/), the Chinese Clinical Trial Registry (ChiCTR) (http://www.chictr.org.cn), and ClinicalTrials.gov (https://clinicaltrials.gov/). In October 2004, an international clinical trial registration platform was established under the leadership of the World Health Organization (WHO). Subsequently, the Chinese Clinical Trial Registry (ChiCTR) was established in 2005. Therefore, January 1, 2005 was selected as the start date of this study. Trials initiated before this date were excluded from the study. The end date of this study was August 1, 2021.

### 2.2 Search strategy and selection criteria

A total of 448,054 clinical trials were registered on the three platforms. The search queries for CDE and ChiCTR were “ischemic stroke” or “cerebral infarction,” and the registration information of 481 clinical trials (114 from CDE and 367 from ChiCTR) relevant to “ischemic stroke” or “cerebral infarction” were manually downloaded. Additionally, we also searched ClinicalTrials.gov with the keyword “ischemic stroke” using the “Condition or disease” field, and specified the country China between the dates January 1, 2005 and August 1, 2021, which identified 365 registered trials. A total of 846 records were identified in the three platforms.

The inclusion criteria for the trials assessed in this study were as follows: (1) The trial focused on ischemic stroke or cerebral infarction; (2) trials were initiated from January 1, 2005 to August 1, 2021; (3) study sites were located in mainland China; and (4) only trials relevant to drug treatment of ischemic stroke were deemed eligible. It was noteworthy that cell therapy and oxygen therapy were also included in this study. The exclusion criteria were as follows: (1) Trials retrospectively registered before January 1, 2005; and (2) trials focusing on predictive or prognostic biomarkers.

**FIGURE 1:**
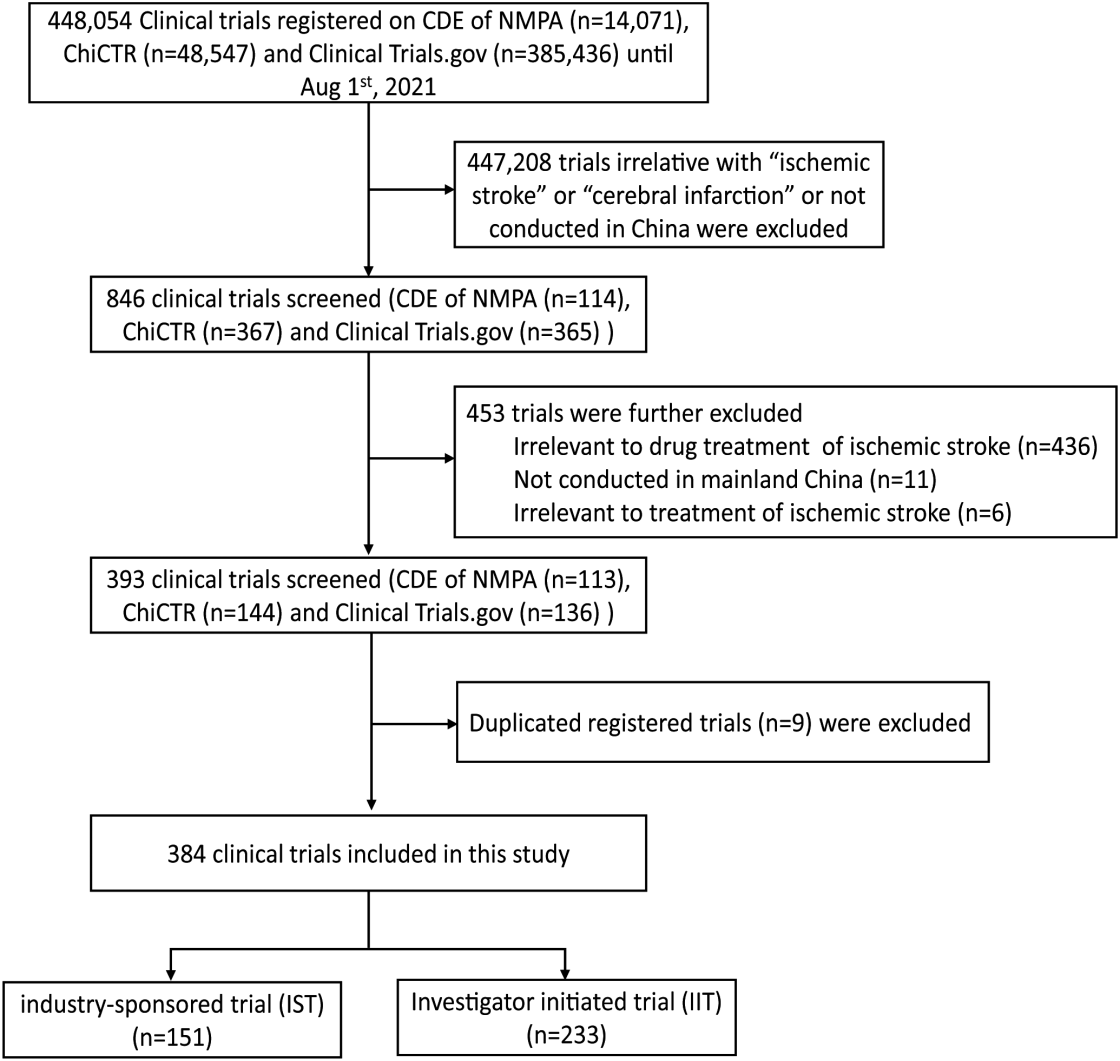
Selection procedure of drug clinical trials for ischemic stroke using three registration platforms

A total of 462 trials were further excluded, encompassing those that did not meet the above criteria, as well as those registered in duplicate. We thus identified 384 drug clinical trials that were associated with “ischemic stroke” and “cerebral infarction” that were initiated between January 1, 2005 and August 1, 2021. Figure 1 shows the selection procedure of this study.

### 2.3 Data extraction and definition

The following information was collected for analysis: the official title of the clinical trial, study design, study type, sponsorship (industry‐sponsored trials (ISTs) or investigator‐initiated trials (IITs)), collaborators, the phase of trial (I–IV, other), newly tested drug, drug type and mechanism of action, date of submission, study locations, geographic region of the leading unit, geographic region of participating centers, number of participating centers, and recruitment status. The registration number was listed in supplementary material and detail information can be searched through the above websites. Only drugs evaluated by ISTs were considered as newly tested drugs. Drug types were classified according to the standard treatment of ischemic stroke. Two investigators (CB and HL) independently extracted data trial by trial from three websites and excluded trials which did not meet above criteria. Then two investigators (CB and HL) identified duplicated trials back‐to‐back. Any discrepancies were discussed and resolved by the consensus of all of the investigators.

### 2.4 Statistical analysis

Descriptive statistics using frequency and percentage was performed to summarize the gathered trial data. We then analyzed the time trend of specific indicators, including the number of trials that were registered, the proportion of trials at each phase, the number of trials based on sponsorship, the geographical distribution of the trials, the number of trials involving different drug types and their corresponding mechanism of action, the number of newly tested drugs, and the number of leading clinical trial units. Chi‐square was used to compare categorical variables. Differences with a two‐sided *p* < 0.05 were considered to be statistically significant. We used the R software (version 4.1.1) in data processing and analysis.

## 3 RESULTS

### 3.1 Overall characters and time trend of all registered drug clinical trials

We identified 384 drug clinical trials that focused on “ischemic stroke” and “cerebral infarction” in three platforms from January 1, 2005 to August 1, 2021 that were conducted in mainland China. In addition, 382 of the 384 (99.5%) clinical trials were domestic, wherein 249 (64.8%) trials were conducted at a single center and 133 (34.6%) by multiple centers. The other two (0.5%) trials were international multiple‐center trials. Furthermore, in terms of the completion status of these 384 clinical trials, 108 (28.1%) were completed, 87 (22.7%) trials have not yet been recruiting, 153 (39.8%) trials are under recruiting, 30 (7.8%) trials are under unknown status and 6 (1.6%) trials are in status of suspension, termination, and withdrawal. In terms of trial types, 166 (43.2%) trials are placebo control trials, 52 (13.5%) trials are head to head trials, 49 (12.8%) trials are one‐arm trials, 96 (25%) trials belong to parallel trials with no placebo, and 21 (5.5%) trials belong to factorial assignment with no placebo. In terms of anticipated enrollment, the number of trials and anticipated enrollment are summarized in Table S1.

**FIGURE 2:**
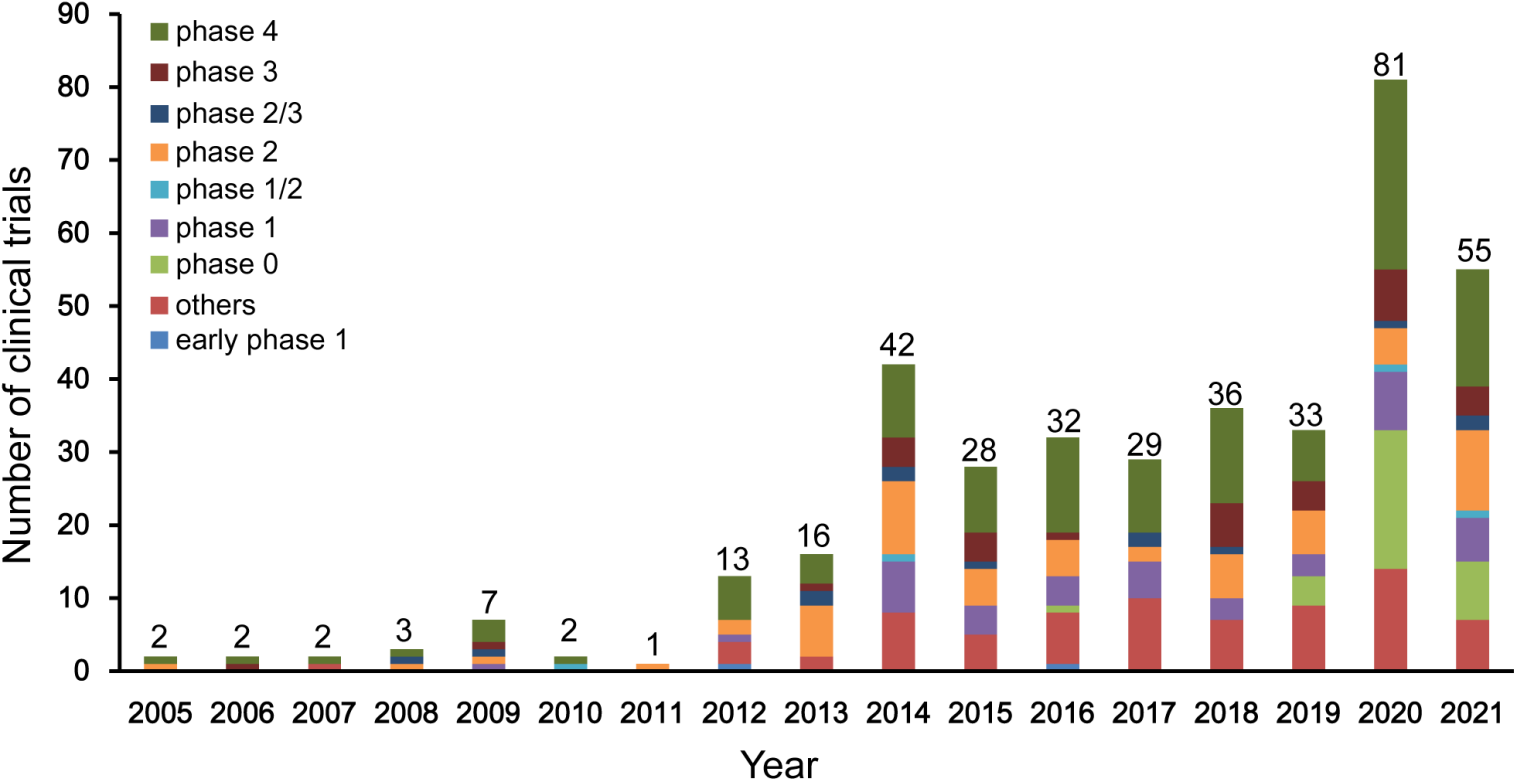
Annual number of clinical trials of ischemic stroke drugs conducted in mainland China based on study phase from 2005 to 2021

From 2005 to 2011, the number of clinical trials was relatively low, although a small peak was observed in 2009 (Figure 2). The number of registered clinical trials increased between 2012 and 2021, with significant increases in 2014 and 2020. The number of registered clinical trials increased from 16 in 2013 to 42 in 2014 and from 33 in 2019 to 81 in 2020, representing an increase of 162.5% and 145.5%, respectively. The number of trials substantially increased from 2012 to 2020, with an average annual growth rate (AAGR) of 39.8%.

In 2015, the State Council of the People's Republic of China established the “Opinions on reforming the examination and approval system for drug and medical devices”,^10^ which is a major policy for the development of clinical trials conducted in mainland China. Therefore, we divided 2005–2021 into two phases: the first phase from 2005 to 2015, and the second phase from 2016 to 2021. Because the data of 2021 were not collected for the entire year, the 2021 data were not included in this investigation. We then analyzed the temporal changes in clinical trial characteristics between the two phases (Table 1). In terms of sponsorship, 49.2% of the trials were ISTs in 2005–2015 compared to 65.8% in 2016–2020 (*p* = 0.003). Furthermore, the percentage of single‐center trials increased from 51.7% in 2005–2015 to 70.7% in 2016–2020 (*p* < 0.001).

**TABLE 1:** Temporal changes in the characteristics of drug clinical trials on ischemic stroke in mainland China

Number of trials (%)					
Characteristic	Total *N* (%)	2005–2015 *N* (%)	2016–2021 *N* (%)	X^2^	*p* Value
Sponsorship					
ISTs	151 (39.3%)	60 (50.8%)	91 (34.2%)	8.7977	0.003016
IITs	233 (60.7%)	58 (49.2%)	175 (65.8%)		
Study phase					
Phase I	42 (10.9%)	13 (11.0%)	29 (10.9%)	8.8229	0.06568
Phase II	63 (16.4%)	28 (23.7%)	35 (13.2%)		
Phase III	33 (8.6%)	11 (9.3%)	22 (8.3%)		
Phase IV	122 (31.8%)	37 (31.4%)	85 (31.9%)		
Others^a^	124 (32.3%)	29 (24.6%)	95 (35.7%)		
Number of centers					
Single center	249 (64.8%)	61 (51.7%)	188 (70.7%)	12.1	0.0005042
Multicenter	135 (35.2%)	57 (48.3%)	78 (29.3%)		

^a^ Others include early phase 1, phase 0, phase 1/2, phase 2/3, and others.

### 3.2 Time trends of clinical trials by study phase

For all 384 trials, 43 (11.2%) trials were observational trials, while 341 (88.8%) trials were interventional trials. In terms of the study phase, 42 (10.9%) trials were phase I, 63 (16.4%) trials were phase II, 33 (8.6%) trials were phase III, while 122 (31.8%) trials were phase IV, with 2 (0.5%), 4 (1.0%), and 13 (3.4%) trials in early phase I, phase I/II, and phase II/III, respectively. In terms of distribution of study phase, phase IV trials were predominant (31.8% [122/384]). Exploratory investigational new drug (eIND) studies, also known as phase 0 clinical trials, accounted for 8.3% [32/384] of all of the trials. Additionally, of the 384 trials, the phase information on 73 (19%) trials was not available at the registered platforms. The proportion of clinical trials classified by study phase was shown in Figure S1. Of the four clinical trial phases (phase I–IV) conducted from 2005 to 2020, phase IV trials showed the greatest growth, followed by phase II, with AAGRs of 27.8% and 17.7%, respectively.

### 3.3 Distribution and time patterns of clinical trials by sponsorship

Based on sponsorship, the number of IITs (60.7% [233/384]) was relatively higher than ISTs (39.3% [151/384]). All of the IITs were registered on ChiCTR or ClinicalTrials.gov platforms. Of the 151 ISTs, 127 (84.1%) trials were domestically sponsored, whereas 24 (15.9%) trials were supported by foreign or multinational pharmaceutical enterprises.

**FIGURE 3:**
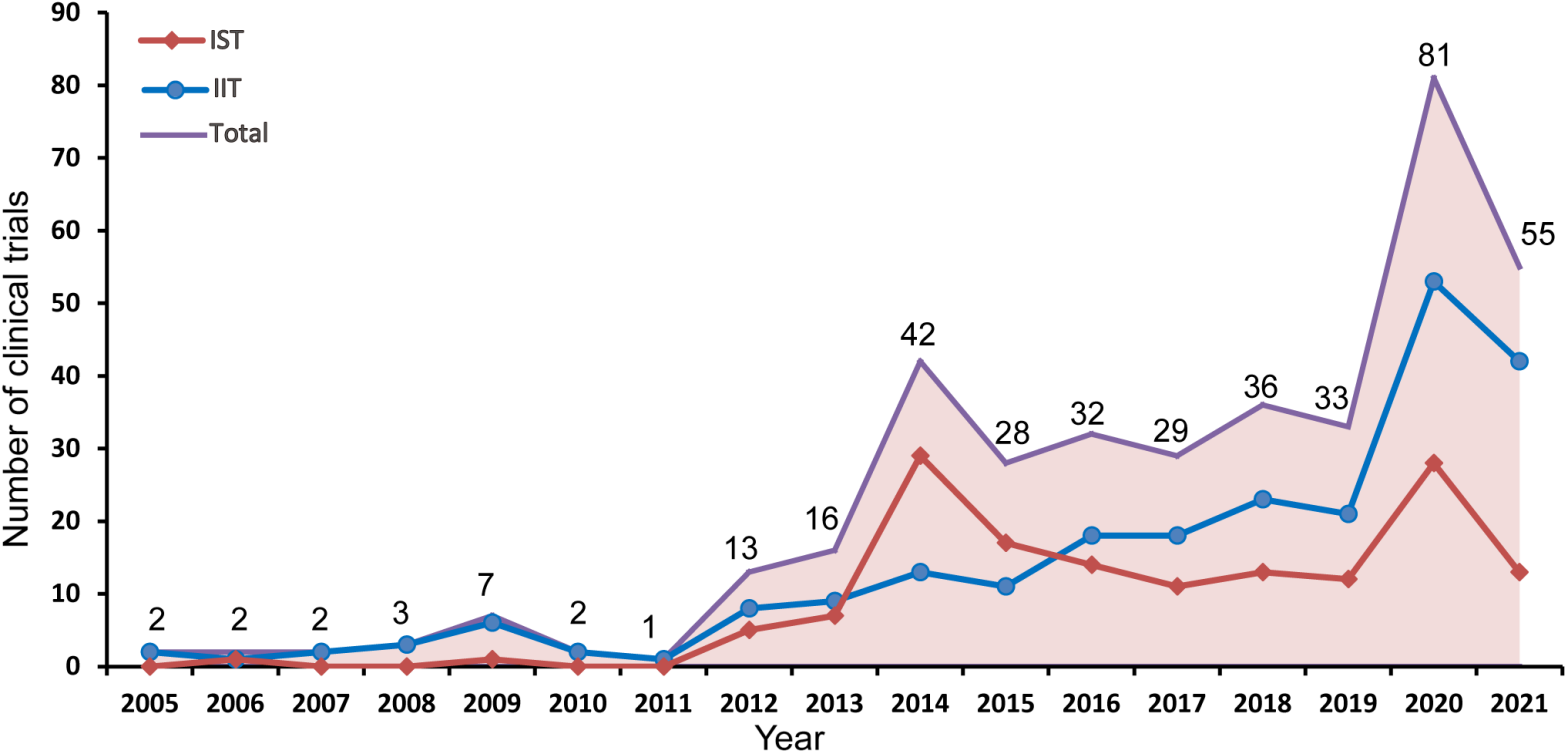
Annual number of ischemic stroke drug clinical trials based on sponsorship in mainland China from 2005 to 2021

In the clinical trials of drugs for the treatment of ischemic stroke in mainland China, the number of IITs gradually increased from 2012 to 2020, with an AAGR of 34.6%. However, there was a notable increase in IIT in 2020, with an annual growth rate of 152.4%. The number of ISTs from 2012 to 2020 was relatively stable, with an AAGR of 52.2%, with two small peaks in 2014 and 2020, representing an annual growth rate of 314.3% and 133.3%, respectively. The peak in the total number in 2014 was mainly due to the sudden increase in ISTs, while the peak in 2020 was due to the combination of IITs and ISTs. In general, IITs were relatively active after 2015 and played a crucial role in drug clinical trials of ischemic stroke in mainland China. Detailed information on the time trends based on sponsorship are shown in Figure 3.

### 3.4 Distribution and time trends of newly tested drugs

**FIGURE 4:**
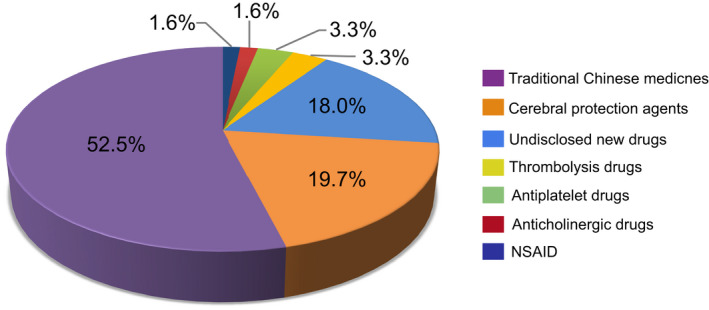
The proportion of newly tested drugs from ISTs during 2005–2021

**FIGURE 5:**
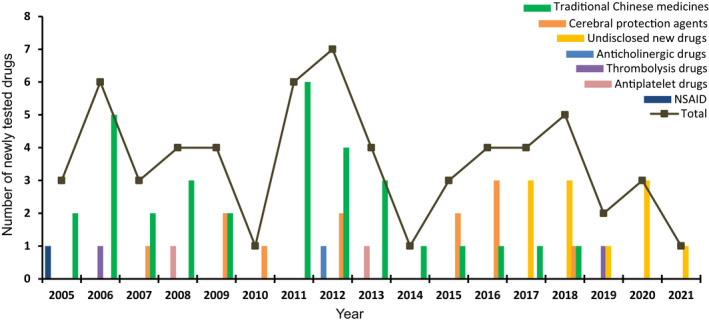
Annual number of newly tested ischemic stroke drugs in ISTs from 2005 to 2021

We further investigated newly tested drugs included in ISTs. From 151 ISTs, a total of 61 newly tested ischemic stroke drugs were investigated in clinical trials between 2005 and 2021. According to the mechanism of action, the newly tested drugs were divided into seven categories, 52.5% (32/61) of drugs were traditional Chinese medicines (TCMs), with 19.7% (12/61) and 18.0% (11/61) of drugs belonging to cerebral protection agents and undisclosed new drugs, respectively. The other four categories were thrombolytic drugs (2/61), antiplatelet drugs (2/61), anticholinergic drugs (1/61), and non‐steroidal anti‐inflammatory drugs (NSAIDs) (1/61). The proportion of 61 newly tested drugs are shown in Figure 4. The number of newly tested drugs in clinical trials showed relatively stable growth trend over time, with an AAGR of 36.8% during 2005–2021 (Figure 5). Newly tested drugs of TCMs emerged almost every year, with notable increases in 2006, 2011, and 2012. However, undisclosed new drugs were included after 2017, suggesting that since 2017, the development of Chinese original‐research drugs significantly increased. The detail information of seven categories of newly tested drugs was summarized in Table S2.

### 3.5 Distribution of clinical trials by drug type or mechanism of action

**FIGURE 6:**
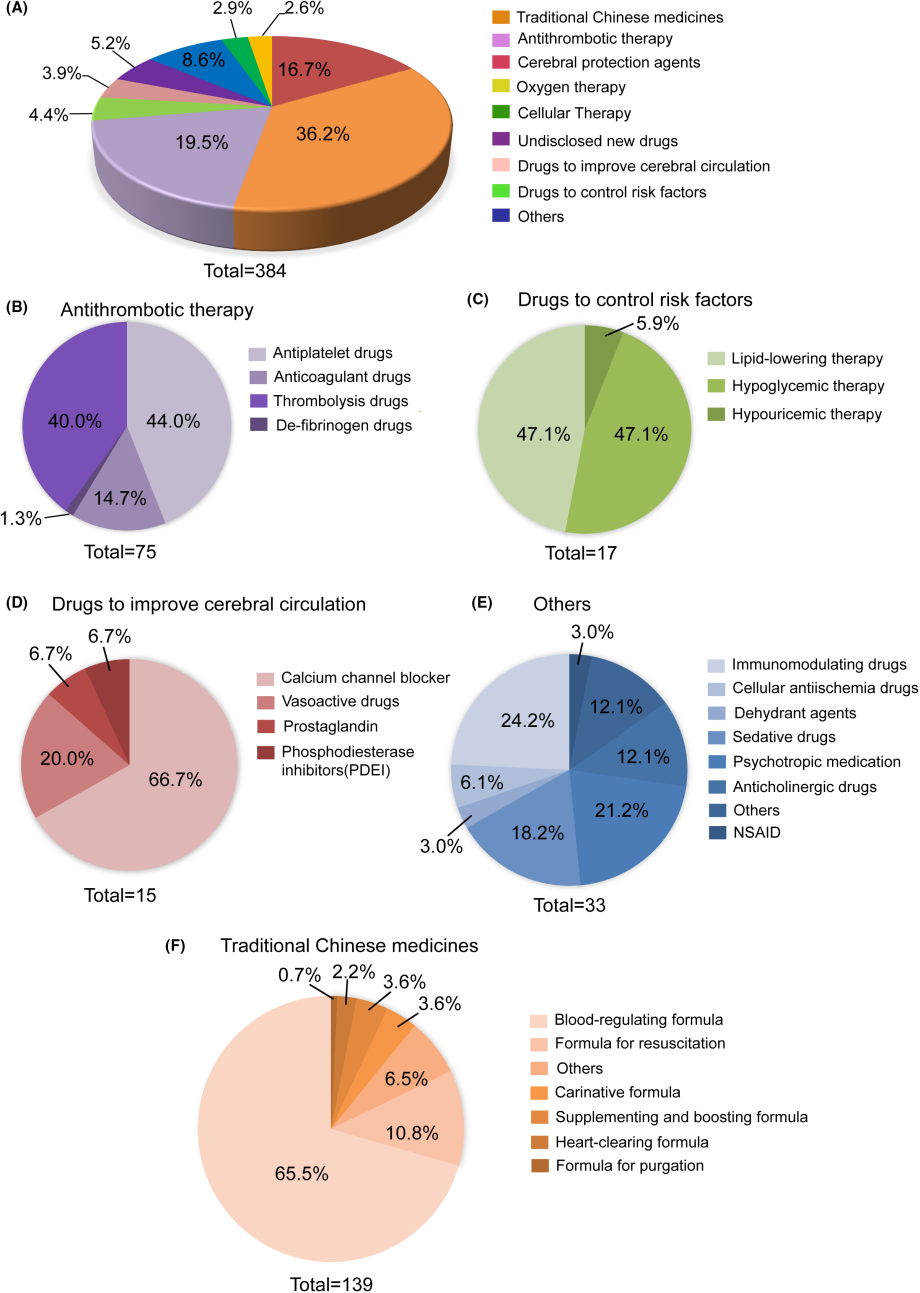
Distribution of clinical trials based on drug type or mechanism of action in mainland China between 2005 and 2021. (A) Overview of clinical trials based on drug type. (B) Distribution of antithrombotic therapeutic trials. (C) Distribution of drug trials on controlling risk factors. (D) Distribution of drug trials for improving cerebral circulation. (E) Distribution of trials involving others, including immunomodulating drugs, cellular anti‐ischemic drugs, dehydrant agents, sedative drugs, psychotropic medication, anticholinergic drugs, non‐steroidal anti‐inflammatory drugs (NSAIDs), and four drugs that cannot be categorized. (F) Distribution of trials involving traditional Chinese medicines (TCMs)

To make it more understandable, we classified the drugs by the drug type and mechanisms of action. The distribution of 384 clinical trials classified by drug type or mechanism of action is shown in Figure 6A. The trials could be divided into nine categories, with TCMs (36.2% [139/384]) as the majority, followed by trials involving antithrombotic therapy (19.5% [75/384]), cerebral protection agents (16.7% [64/384]), undisclosed new drugs (5.2% [20/384]), drugs to control risk factors (4.4% [17/384]), drugs to improve cerebral circulation (3.9% [15/384]), cellular therapy (2.9% [11/384]), oxygen therapy (2.6% [10/384]), and others (8.6% [33/384]).

In terms of antithrombotic therapy, antiplatelet drugs (44.0% [33/75]) and thrombolytic drugs (40% [30/75]) accounted for the majority of antithrombotic drugs. In addition, there were also anticoagulant drugs (14.7% [11/75]) and defibrinogen drugs (1.3% [1/75]) (Figure 6B). The detail information of clinical trials associated with antithrombotic drugs was summarized in Table S3. Seventeen of the 384 clinical trials were associated with controlling risk factors of ischemic stroke, which could be divided into three subcategories: lipid‐lowering therapy (47.1% [8/17]), hypoglycemic therapy (47.1% [8/17]), and hypouricemic therapy (5.9% [1/17]) (Figure 6C). Fifteen trials were related to drugs that improve cerebral circulation, among which 66.7% (10/15) were calcium channel blockers, 20% (3/15) were vasoactive drugs, and prostaglandin (1/15) and phosphodiesterase inhibitors (PDEI) (1/15) accounted for 6.7%, respectively (Figure 6D).

In addition, there were several special drugs, including immunomodulating drugs (*n* = 8), cellular anti‐ischemia drugs (*n* = 2), dehydrant agents (*n* = 1), sedative drugs (*n* = 6), psychotropic medication (*n* = 7), anticholinergic drugs (*n* = 4), non‐steroidal anti‐inflammatory drugs (NSAIDs) (*n* = 1), and uncategorized drugs (*n* = 4) (Figure 6E).

The largest proportion of all of the clinical trials comprised TCMs, suggesting potential treatment strategies for ischemic stroke. The TCMs were categorized into seven subtypes according to Chinese pharmacopeia: blood‐regulating formulae (65.5% [91/139]), formulae for resuscitation (10.8% [15/139]), carinative formulae (3.6% [5/139]), supplementing and boosting formulae (3.6% [5/139]), heart‐clearing formulae (2.2% [3/139]), formulae for purgation (0.7% [1/139]), and other uncategorized agents (6.5% [9/139]) (Figure 6F).

Furthermore, besides TCMs, 49 out of 384 clinical trials did not merely study just one drug, they had combinations of different types of drugs. The majority of trials focused on antithrombotic therapy (65.3%[32/49]), others included cerebral protection (10.2%[5/49]), cerebral protection in combination with antithrombotic therapy (10.2%[5/49]), sedative drugs (6.1%[3/49]), lipid‐lowering therapy (4.1%[2/49]), hypoglycemic therapy(2.0%[1/49]) and dehydrant agent (2.0%[1/49]). Detail information about clinical trials of combination drug types was summarized in Table S4.

### 3.6 Geographical distribution of drug clinical trials

**FIGURE 7:**
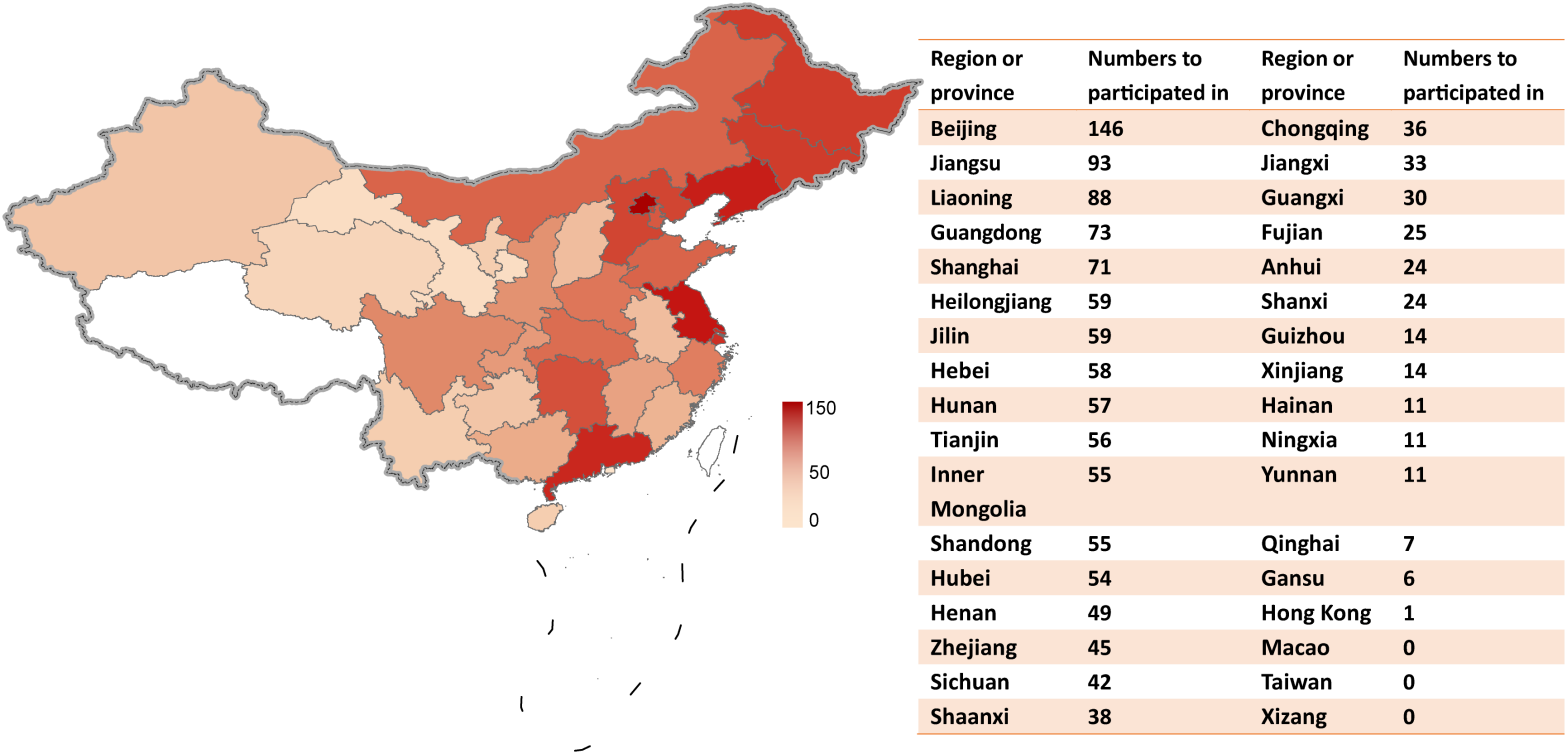
Geographical distribution of trials according to provinces or municipalities that participated in across mainland China in 2005–2021

**FIGURE 8:**
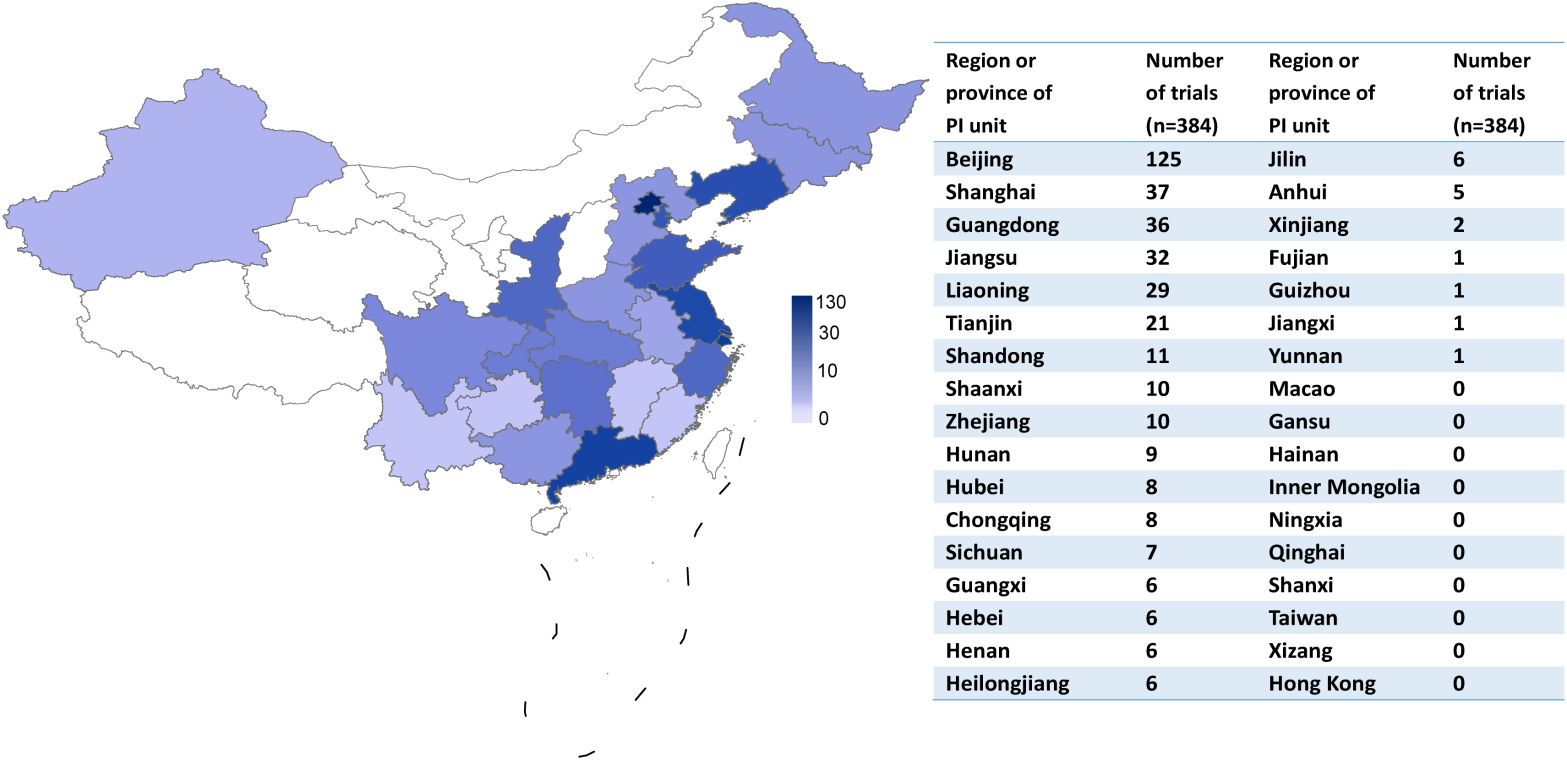
Geographical distribution of drug trials based on Chinese provinces or municipalities of the principal investigator from 2005 to 2021

Overall, 384 clinical trials of the ischemic stroke drugs were conducted in 31 different provinces or municipalities in China (Figure 7). Of these, Beijing (*n* = 146), Jiangsu (*n* = 93), Liaoning (*n* = 88), Guangdong (*n* = 73), and Shanghai (*n* = 71) were the top five provinces or municipalities that participated in the clinical trials. In terms of province or municipalities where the principal investigators (PIs) are located (Figure 8), 238 of 384 (62.0%) clinical trials were conducted in Beijing, followed by Shanghai (*n* = 37), Guangdong (*n* = 36), Jiangsu (*n* = 32), and Liaoning (*n* = 29). In contrast, the number of trials headed by PIs from Xinjiang (*n* = 2), Fujian (*n* = 1), Guizhou (*n* = 1), Jiangxi (*n* = 1), and Yunnan (*n* = 1) was less than five. Drug clinical trials on ischemic stroke in China show an uneven geographical distribution (Figures 7 and 8).

### 3.7 Time trends of leading clinical trial units

The temporal patterns of newly added leading clinical trials between 2005 and 2021 were further assessed (Figure S2). Of all 384 clinical trials, four trials lost leading unit information. The number of leading drug clinical trial units on ischemic stroke conducted in mainland China increased, with a total of 145 by August 1, 2021. In addition, the number of newly added leading drug clinical trial units was relatively low, reaching only 10 by 2011. Since 2012, the number began to increase, and substantial growths were observed in 2014 and 2020, at 23 and 37, respectively. The trend of leading units was consistent with the trend of all of the clinical trials shown in Figure 2.

## 4 DISCUSSION

Stroke is a chronic disease and a major public health concern in China. Ischemic stroke is the predominant type of stroke, accounting for 69.6%–70.8% of stroke cases in the country and is the leading cause of death and disability among adults in China.^11^, ^12^ Therapeutic paradigms have evolved over the past two decades, driven primarily by the development of antithrombotic therapy, neuroprotective therapy, and cholesterol‐lowering therapy, and have significantly improved treatment schemes for ischemic stroke.^13^ Advances in ischemic stroke therapeutics are largely attributable to novel drugs and swift clinical trial development. At the same time, a large number of reviews have reviewed the current status of drug clinical trials for ischemic stroke from different perspectives both abroad and domestically. For example, some reviews focused on the overall presentation of drug clinical trials for ischemic stroke,^14^, ^15^, ^16^ while some other reviews focused on specific types of drugs or strategies, such as neuroprotective strategies,^17^ modulation of neuroinflammation,^18^ stem cell therapies,^19^ tenecteplase,^20^ rapamycin,^21^ erythropoietin, and its derivatives,^22^ etc. However, no investigations on the evolution of ischemic stroke drug clinical trials in mainland China have been conducted to date. Therefore, this serves as the first study to comprehensively assess the overall temporal profiles of drug clinical trials on ischemic stroke in mainland China.

The rapid increase in the number of drug clinical trials and R&D activities on innovative drugs for the treatment of ischemic stroke is largely attributable to the policies initiated by the Chinese government.^23^ Since 2008, the Chinese government has launched national major projects for new drugs by improving the procedure of review and approval process. By 2015, the Chinese government established several reform policies^10^, ^24^ that prioritize the review process for innovative drugs. By 2017, the NMPA had become a regular member of the International Council for Harmonization (ICH).^25^, ^26^ Together, these efforts have facilitated in the development of new drugs as well as drug clinical trials for ischemic stroke.

Furthermore, since 2009, the Chinese government has come up with a series of policies to promote the development of stroke prevention and treatment in China: the Stroke Screening and Prevention Project was launched in 2009, the National Stroke Prevention and Control Engineering Committee was established in 2011, the “China Stroke Center Construction Standards” was formulated in 2012, the China National Stroke Screening Survey (CNSSS) was established in 2013, the construction of stroke centers was initiated in 2015, and the Stroke Emergency Map was promoted in 2018.^11^, ^12^, ^27^, ^28^ We surmise that the increase in the number of ischemic stroke clinical trials in 2014 may also be related to the establishment of the CNSSS program in 2013. The introduction of these policies also improved the treatment of stroke and accelerated the speed of drug development and clinical trials for ischemic stroke to a certain extent.

TCMs comprise the largest proportion (54%) of newly tested drugs in ISTs (Figure 4). An increase in the number of newly tested drugs occurred in 2015–2021 (Figure 5). In 2006 and 2011, the number of newly tested drugs developed in TCM was the highest. In addition, TCMs also compromised the largest proportion (36.2%) of all of the clinical trials (Figure 6A). Besides, on November 3, 2015, CDE issued the “Technical Guidelines for Clinical Research on New Traditional Chinese Medicine in the Treatment of Stroke”.^29^ This served as the regulatory policy hiatus for TCM clinical trials for ischemic stroke. At the same time, the rate of R&D of new TCMs has stabilized since 2016 (Figure 5). However, TCM is not the main treatment method for ischemic stroke, especially in hyperacute period. In this study, the clinical trials classified as TCM group may not only receive TCM treatment, but also combine with other interventional therapies for ischemic stroke.

Another hotspot is antithrombotic therapy, which accounted for 19.5% in clinical trials for ischemic stroke, is one of the most important cornerstones for the treatment of ischemic stroke. Antiplatelet drugs and thrombolytic drugs account for 44% and 40% in antithrombotic therapies, respectively (Figure 6B). The clinical application of tPA is a milestone in the treatment of AIS and has changed the status of neurology in the treatment of AIS. At present, there are three generations of drugs that are commonly used in clinical thrombolytic therapy. The first generation includes streptokinase (SK) and urokinase (uPA).^30^, ^31^ The main representative of the second generation of thrombolytic drug is Alteplase (rtPA), which was first approved by the Food and Drug Administration (FDA) for the treatment of AIS.^32^ The third generation of clinically used thrombolytic drugs are mainly rtPA mutants (tenecteplase and reteplase, etc).^13^, ^20^ The domestic third‐generation thrombolytic drugs were also actively being developed in China. Clinical trials associated with tenecteplase accounted for 40% (12/30) of the thrombolysis drugs. In 2018, a domestic tenecteplase (recombinant human TNK tissue‐type plasminogen activator for injection, rhTNK‐tPA, Mingfule®) phase II clinical trial (TRACE study) was conducted to explore the safe and effective dose range for hyper‐acute ischemic stroke in Chinese patients,^33^ and the drug and was approved by NMPA on November 13, 2019.

At the same time, the possibility of antiplatelet drugs in the treatment of ischemic stroke was explored in China. Because antiplatelet drugs are mostly developed drugs, IITs accounted for 75.8% (25/33) of the clinical trials on antiplatelet drugs. Most investigators have been searching better methods and combinations of antiplatelet drugs for treating ischemic stroke. For example, CHANCE (clopidogrel with aspirin in acute minor stroke or transient ischemic attack)^34^ was the first Chinese clinical trial that had rewritten international guidelines.^35^, ^36^ In addition, clinical trials of other types of antiplatelet drugs such as adenosine diphosphate (ADP) receptor antagonist (ticagrelor)^37^, ^38^ and glycoprotein IIb/IIIa (GPIIb/IIIa) receptor antagonist (tirofiban)^39^ were also undergoing or completed, which revealed new information on the treatment of ischemic stroke.

Except for TCMs, the R&D of new drugs for ischemic stroke mainly focus on thrombolysis and neuroprotective therapy, which is based on the “Technical guidelines for clinical trials of therapeutic drugs for acute ischemic stroke” that was issued on February 9, 2018 by the CDE of NMPA.^29^ The clinical trials of cerebral protection agents accounted for 16.7% (64/384) of all clinical trials (Figure 6A) and 19% of the newly tested drugs (Figure 4). In addition, most undisclosed new drugs were reported to be cerebral protection drugs; however, detailed information is unavailable. Among the cerebral protection drugs, butylphthalide (NBP) is the most commonly tested drug in clinical trials [31.3% (20/64)]. In addition, other types of drugs that take part in the protection of the brain continue to be explored, including free radical scavengers edaravone, and anti‐oxidative agents nitrate ketone oxazine, N‐methyl‐D‐aspartate receptor (NMDAR) antagonist sofadil, and human urinary kallidinogenase,^40^ etc.

Although substantial progress has been made in drug clinical trials for ischemic stroke over the past 17 years, there are still some concerns that need to be addressed. First, the leading clinical trial units in mainland China show heterogeneous geographical distribution (Figure 8), indicating that more resources are allocated to leading hospitals in Beijing, Shanghai, Guangdong, Jiangsu, and Liaoning. However, units participating in clinical trials across China showed a better distribution (Figure 7). Northeastern China shows active engagement, reflecting a large population of patients with ischemic stroke. These results may appeal to clinicians, policy makers in areas with a high incidence of ischemic stroke to pay more attention to ischemic stroke. Next, there are multiple clinical trials for drugs of the same type and mechanism of action, particularly TCMs. This is because of the low risk and low cost of generic drugs. However, clinical trials on generic drugs are also largely duplicated and thus may be a waste of resources, hindering the development of original innovative drugs.

This study has some limitations. First, there may be potential selection bias when manually searching all drug clinical trials for “ischemic stroke” and “cerebral infarction”. Second, the data we collected began on January 1, 2015, but the WHO International Clinical Trial Registration Platform and the Declaration of Helsinki (2008) only required mandatory registration of clinical trials in 2007. Therefore, some clinical trials that ended before 2007 without retrospective registration may have been omitted. Third, owing to the complex mechanism involved in cerebral protection drugs in China, we did not conduct further classification analysis of these drugs. Forth, some data such as drug type and phase of study were not provided by the registration platforms. Fifth, the data of this study was retrieved from three online clinical trial registry platforms. Not all of the outcomes of clinical trials included in our study were published. Thus, the information including treatment effect and actual number of participants and the severity of the degree, cannot be fully collected and analyzed. Last, the inclusion criteria, exclusion criteria, primary outcomes, and secondary outcomes of each clinical trial contain many contents and items, with different categories. For example, outcomes of phase I clinical trials included maximum plasma concentration (Cmax), time to maximum plasma concentration (Tmax), area under the curve (AUC), elimination half‐life (T1/2), volume of distribution (Vd), and various laboratory evaluation. However, most of phase 2–4 clinical trials focused on clinical function score (National Institute of Health Stroke Scale (NIHSS), modified Rankin scale (mRS), Barthel Index (BI), Activity of Daily Living (ADL) Scale, Glasgow Coma Scale (GCS), Mini‐mental State Examination (MMSE), Montreal Cognitive Assessment (MoCA) score, etc.) and radiological indicators (lesion volume, hemorrhage volume, etc.). Other outcomes included new stroke events, new clinical vascular events, adverse events, all‐cause death. Besides, The Scale of Traditional Chinese Medicine (SSTCM) will also be evaluated when referring to clinical trials about TCMs. So, there is no way to do a uniform consistency analysis.

## 5 CONCLUSION

In conclusion, this study presents an overview of clinical trials of drugs for the treatment of ischemic stroke from 2005 to 2021 in mainland China. The drug clinical trials and new drug R&D efforts on ischemic stroke have rapidly improved in the past 17 years, thereby providing more treatment options for Chinese ischemic stroke patients. The prevention and treatment of ischemic stroke in China remain severe, and thus the exploration of innovative drugs for ischemic stroke should be strengthened.

## CONFLICT OF INTEREST

The authors declare that they have no known competing financial interests or personal relationships that could have appeared to influence the work reported in this paper.

## AUTHOR CONTRIBUTIONS

CRB and HYL contributed to the study concept and design. CRB, HXZ, and LXC collected the data. TQW, JMH, and CRB were involved in the interpretation of data. CRB and CBH analyzed the data. CRB visualized the data. CRB and HYL drafted the original manuscript. CRB and LHW revised and edited the manuscript. All authors read and approved the final manuscript.

## Supporting information


Figure S1

Figure S2

**Appendix S1** Supporting Information

Table S1

Table S2

Table S3

Table S4

## Data Availability

The data that support the findings of this study are available from the corresponding authors upon reasonable request.
