# Supplementary figures and images for: Evolution of ischemic stroke drug clinical trials in mainland China from 2005 to 2021
Source: CNS Neurosci Ther. 2022 Jun 1;28(8):1229–39. doi: 10.1111/cns.13867 (PMC9253749; doi:10.1111/cns.13867)

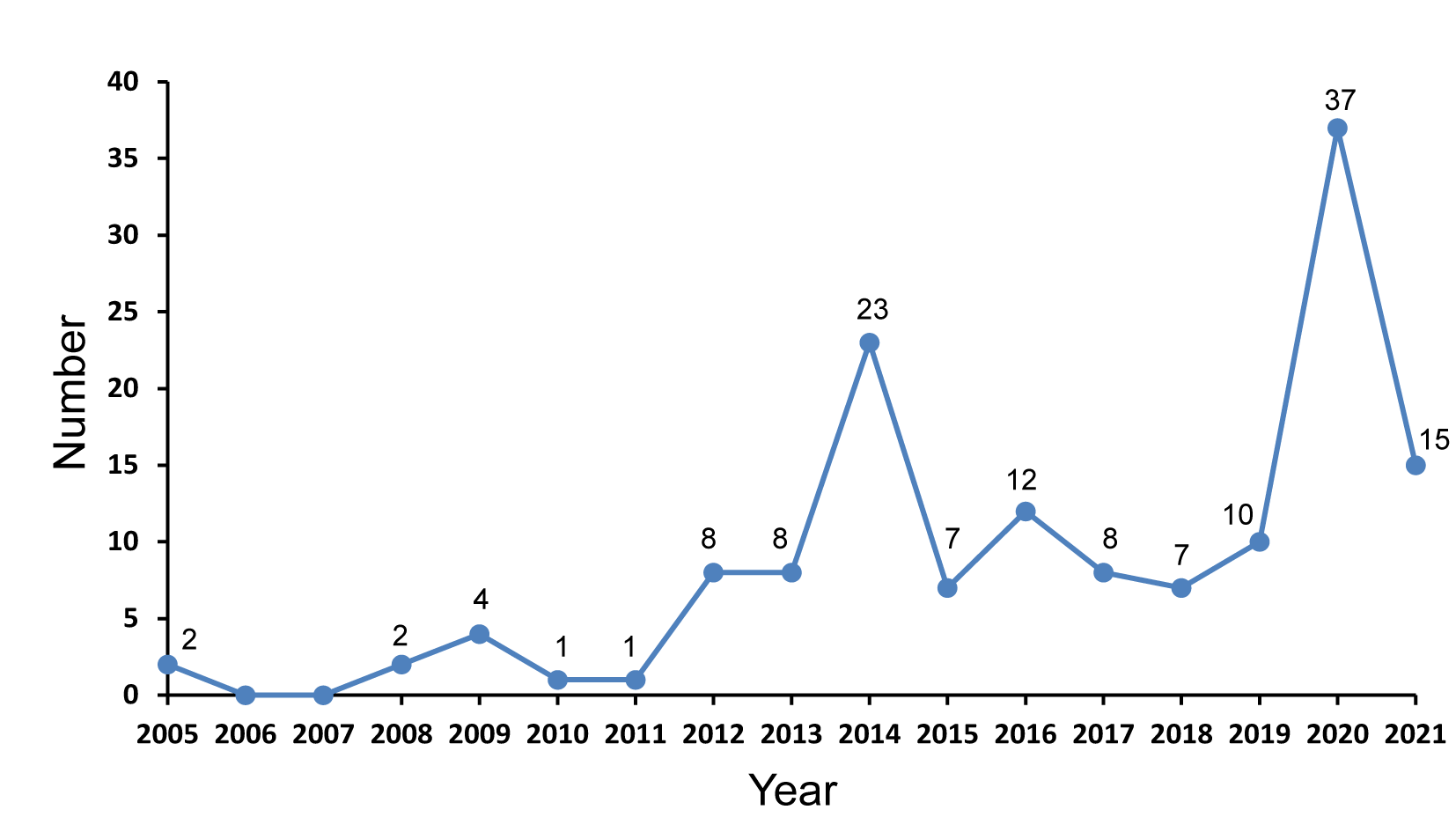

Supplement: Supplementary file 2 — Figure S2 [file CNS-28-1229-s007.tif]
