# Supplementary material for: Evolution of ischemic stroke drug clinical trials in mainland China from 2005 to 2021
Source: CNS Neurosci Ther. 2022 Jun 1;28(8):1229–39. doi: 10.1111/cns.13867 (PMC9253749; doi:10.1111/cns.13867)
Supplement: Supplementary file 4 — Table S1 [file CNS-28-1229-s006.docx]

**Table S1. Summarization of anticipated enrollment of ischemic stroke drug clinical trials.**

| **Anticipated enrollment** | **Number of clinical trials** | **Percentage** |
| --- | --- | --- |
| 0-50 | 62 | 16.1% |
| 51-100 | 69 | 18.0% |
| 101-200 | 71 | 18.5% |
| 201-300 | 50 | 13.0% |
| 301-400 | 24 | 6.3% |
| 401-500 | 18 | 4.7% |
| 501-1000 | 38 | 9.9% |
| 1001-2000 | 17 | 4.4% |
| 2001-5000 | 18 | 4.7% |
| 5001-10000 | 6 | 1.6% |
| ＞10000 | 4 | 1.0% |
| No data | 7 | 1.8% |
