# Supplementary material for: Evolution of ischemic stroke drug clinical trials in mainland China from 2005 to 2021
Source: CNS Neurosci Ther. 2022 Jun 1;28(8):1229–39. doi: 10.1111/cns.13867 (PMC9253749; doi:10.1111/cns.13867)
Supplement: Supplementary file 5 — Table S2 [file CNS-28-1229-s005.docx]

**Table S2. Detail information of seven categories of 61 newly tested drugs.**

| **Drug type** | **Drug Chinese name** | **Drug English name** |
| --- | --- | --- |
| NSAID | 铝镁司片(II) | Compound Aspirin Tablets |
| Anticholinergic drugs | 左旋盐酸苯环壬酯片 | L-phencynonate hydrochloride tablets |
| Antiplatelet drugs | 三氟柳胶囊 | Triflusal capsules |
|  | 吡拉格雷钠注射液 | Pyragrel Sodium for Injection |
| Cerebral protection agents | 恩必普氯化钠注射液 | Butylphthalide and Sodium Chloride Injection |
|  | 注射用硝酮嗪 | Nitrate ketone oxazine |
|  | 复方依达拉奉注射液 | Compound Edaravone Injection |
|  | 注射用匹诺塞林 | Pinocembrin for Injection |
|  | 安脑三醇 | YC-6 |
|  | 索法地尔 | Sofadil (Neu2000KWL) |
|  | Y-2舌下片 | Y-2 sublingual tablets |
|  | 羟戊基苯甲酸钾片 | Potassium-Hydroxy pentyl Benzoate tablets |
|  | 注射用羟戊基苯甲酸钾 | Potassium-Hydroxy pentyl Benzoate for Injection |
|  | 注射用吗利福肽 | Monocyte Locomotion Inhibitory Factor for Injection |
|  | 注射用布罗佐喷钠 | Sodium(±)-5-Bromo-2-(α-hydroxypentyl) benzoate (BZP) |
|  | 牛磺酸氯化钠注射液 | Taurine sodium chloride injection |
| Thrombolytic drugs | 注射用重组人尿激酶原 | rhPro-uk |
|  | 替奈普酶 | Tenecteplase |
| Undisclosed new drugs | SY-007 | SY-007 |
|  | B1101 | B1101 |
|  | BJY-801 | BJY-801 |
|  | HY-021068 | HY-021068 |
|  | QHRD106 | QHRD106 |
|  | SPT-07A | SPT-07A |
|  | TFZ16 | TFZ16 |
|  | XY03-EA | XY03-EA |
|  | GD-11 | GD-11 |
|  | KPCXM18 | KPCXM18 |
|  | LT3001 | LT3001 |
| Traditional Chinese medicines (TCMs) | 葛酮通络胶囊 | Getong Tongluo capsule |
|  | 华佗再造丸 | Huatuo Zaizao pills |
|  | 蒺藜皂苷胶囊 | Tribulus Terrestris Saponin capsule |
|  | 龙血通络胶囊 | Longxue Tongluo capsule |
|  | 银杏内酯葡胺注射液 | Ginkgolides Meglumine Injection |
|  | 参丹通脑滴丸 | Shendan Tongnao dropping pill |
|  | 赤苷脉通胶囊 | Erythrin Maitong capsule |
|  | 灯盏丹芪胶囊 | Dengzhan Danqi capsule |
|  | 复脑素注射液 | Compound encephalin injection |
|  | 海伦胶囊 | Helen capsule |
|  | 活络通脑片 | Huoluotongnao tablet |
|  | 七叶通脉胶囊 | Qiye Tongmai capsule |
|  | 舒脑欣滴丸 | Shunaoxin dropping pill |
|  | 天龙通络胶囊 | Tianlong Tongluo capsule |
|  | 通脑溶栓胶囊 | Tongnao thrombolytic capsule |
|  | 醒脑舒络片 | Xingnao Shuluo tablet |
|  | 醒神化瘀滴丸 | Xingshen Huayu dropping pill |
|  | 血塞通肠溶胶囊 | Xuesaitong enteric capsule |
|  | 血塞通肠溶片 | Xuesaitong enteric-coated tablet |
|  | 血元通颗粒 | Xueyuantong granules |
|  | 银杏内酯B注射液 | Ginkgolide B injection |
|  | 银杏内酯滴丸 | Ginkgolide dropping pill |
|  | 银杏内酯注射液 | Ginkgolide injection |
|  | 银杏总内酯滴丸 | Ginkgo total lactone dropping pill |
|  | 银杏总内酯胶囊 | Ginkgo total lactone capsule |
|  | 蛭龙通络片 | Zhilong Tongluo tablet |
|  | 中风回语颗粒 | Stroke anaphora granules |
|  | 注射用丹参多酚酸 | Salvianolic acid for injection |
|  | 注射用海参糖胺聚糖 | Holothurian glycosaminoglycan for injection |
|  | 注射用甲磺酸胺银内酯B | Simethylaminoethyl ginkgolide B mesylate for injection |
|  | 注射用三花粉针剂 | Sanhua Powder Injection |
|  | 注射用银杏达莫 | Ginkgo Leaf Extract and Dipyridamole Injection |
