# Supplementary material for: Evolution of ischemic stroke drug clinical trials in mainland China from 2005 to 2021
Source: CNS Neurosci Ther. 2022 Jun 1;28(8):1229–39. doi: 10.1111/cns.13867 (PMC9253749; doi:10.1111/cns.13867)
Supplement: Supplementary file 6 — Table S3 [file CNS-28-1229-s001.docx]

**Table S3. Detail information of antithrombotic drugs.**

| **Database** | **Number** | **Title** | **Status** | **Drug type** | **Interventions** | **Phase** | **Enrollment** | **IIT/IST** | **Study Type** | **Start year** | **Start Date** | **Single center or multiple center** |
| --- | --- | --- | --- | --- | --- | --- | --- | --- | --- | --- | --- | --- |
| ChiCTR | ChiCTR2100045812 | 阿加曲班联合双重抗血小板治疗超溶栓时间窗分水岭脑梗死的疗效和安全性评价：一个多中心、前瞻性、开放标签的随机对照临床试验 | Recruiting | anticoagulant drugs | Argatroban\|Aspirin\|Clopidogrel | Phase 4 | 240 | IIT | Interventional | 2021 | 2021/4/25 | single center |
| ChiCTR | ChiCTR-IPR-16008663 | 阿加曲班对急性缺血性脑卒中基底静脉回流及预后的影响 | Recruiting | anticoagulant drugs | Argatroban | Phase 0 | 60 | IIT | Interventional | 2016 | 2016/6/17 | multiple center |
| ChiCTR | ChiCTR-OPN-16007931 | 肝素、双抗（波立维及阿司匹林）及单抗（阿司匹林）治疗急性期脑梗死及TIA的临床研究 | Recruiting | anticoagulant drugs | Heparin\|Aspirin\|Clopidogrel | Phase 4 | 588 | IIT | Interventional | 2016 | 2016/2/16 | single center |
| ChiCTR | ChiCTR1800020026 | 脑梗死并G6PD缺乏者阿司匹林抗血小板治疗有效性和安全性的多中心前瞻性随机对照研究 | Pending | antiplatelet drugs | Aspirin | Phase 4 | 400 | IIT | Interventional | 2018 | 2018/12/12 | multiple center |
| ChiCTR | ChiCTR1900025936 | 替罗非班治疗非溶栓脑梗死及短暂性脑缺血发作病例的临床疗效和安全性：一项基于病例数据库的真实世界回顾性研究 | Pending | antiplatelet drugs | Tirofiban | Not Applicable | 440 | IIT | observational | 2019 | 2019/9/15 | multiple center |
| ChiCTR | ChiCTR2000030824 | 阿司匹林、氯吡格雷联合丁苯酞对中老年急性缺血性脑卒中患者精神、神经和行为功能的影响 | Recruiting | antiplatelet drugs | Aspirin\|ClopidogrelButyphthalide | Phase 0 | 200 | IIT | Interventional | 2020 | 2020/3/15 | single center |
| ChiCTR | ChiCTR2000031297 | 替罗非班对急性缺血性脑卒中患者临床疗效和安全性研究 | Recruiting | antiplatelet drugs | Tirofiban | Phase 4 | 400 | IIT | Interventional | 2020 | 2020/3/27 | single center |
| ChiCTR | ChiCTR2100042628 | 血小板活化因子受体拮抗剂对脑梗死急性期疗效评价及其抗栓及抗炎神经保护作用的研究 | Recruiting | antiplatelet drugs | Platelet activating factor receptor antagonist | Not Applicable | 200 | IIT | Interventional | 2021 | 2021/1/24 | single center |
| ChiCTR | ChiCTR-IOR-14005295 | 急性脑梗死溶栓后根据血栓弹力图选择性早期联用替罗非班的安全性及有效性评估 | Recruiting | antiplatelet drugs | Tirofiban | Not Applicable | 120 | IIT | Interventional | 2014 | 2014/9/29 | single center |
| ChiCTR | ChiCTR-IPR-15006826 | 双抗和降纤治疗急性大动脉粥样硬化性脑梗死的随机、平行对照、多中心临床比较研究 | Recruiting | antiplatelet drugs | Aspirin\|Clopidogrel\|Defibrinogen | Phase 4 | 400 | IIT | Interventional | 2015 | 2015/7/28 | single center |
| ChiCTR | ChiCTR-OCH-12002681 | 基因多态性对氯吡格雷治疗急性脑梗死有效性影响的研究 | Completed | antiplatelet drugs | Clopidogrel | Not Applicable | 300 | IIT | observational | 2012 | 2012/11/12 | single center |
| ChiCTR | ChiCTR-OCH-12002951 | 基因多态性对阿司匹林治疗急性脑梗死有效性影响的研究 | Completed | antiplatelet drugs | Aspirin | Not Applicable | 300 | IIT | observational | 2012 | 2012/12/30 | single center |
| ChiCTR | ChiCTR-OIN-17013510 | 替罗非班在非大血管闭塞急性脑梗死患者中安全性及有效性研究 | Recruiting | antiplatelet drugs | Tirofiban | Phase 2 | 100 | IIT | observational | 2017 | 2017/11/23 | single center |
| ChiCTR | ChiCTR-ONN-17013511 | 急性脑梗死患者动脉内治疗围手术期使用替罗非班干预的安全性及有效性研究 | Recruiting | antiplatelet drugs | Tirofiban | Not Applicable | 160 | IIT | Interventional | 2017 | 2017/11/23 | multiple center |
| ChiCTR | ChiCTR-OCH-14004480 | 巴曲酶注射液（东菱迪芙）治疗进展性脑梗死的临床研究 | Recruiting | defibrinogen drugs | Batroxobin | Phase 4 | 400 | IIT | observational | 2014 | 2014/4/8 | multiple center |
| ChiCTR | ChiCTR1800014666 | 低剂量rt-PA联合替罗非班治疗 非心源性急性脑梗死的临床疗效研究 | Recruiting | thrombolytic drugs | Alteplase+Tirofiban | Phase 2 | 100 | IIT | Interventional | 2018 | 2018/1/28 | single center |
| ChiCTR | ChiCTR1800016519 | 注射用重组人尿激酶原（rhPro-uk）静脉溶栓治疗急性缺血性脑卒中Ⅱa期临床研究 | Completed | thrombolytic drugs | rhPro-uk | Phase 2 | 200 | IST | Interventional | 2018 | 2018/6/6 | multiple center |
| ChiCTR | ChiCTR1900024108 | 超时间窗应用尿激酶对急性脑梗死疗效的临床研究 | Recruiting | thrombolytic drugs | Urokinase | Phase 0 | 120 | IIT | observational | 2019 | 2019/6/26 | multiple center |
| ChiCTR | ChiCTR2000029023 | 阿加曲班联合阿替普酶治疗急性脑梗死的有效性和安全性研究 | Recruiting | thrombolytic drugs | Argatroban+alteplase | Phase 0 | 100 | IIT | Interventional | 2020 | 2020/1/11 | single center |
| ChiCTR | ChiCTR2000029556 | 低剂量rt-PA联合替罗非班治疗DWI-Flair不匹配的发病时间不明的急性非心源性脑梗死的临床安全及疗效 | Not yet recruiting | thrombolytic drugs | alteplase+Tirofiban | Phase 0 | 80 | IIT | Interventional | 2020 | 2020/2/4 | single center |
| ChiCTR | ChiCTR2000034607 | 阿替普酶（rTPA）溶栓治疗急性脑梗死患者神经功能变化的影响因素研究 | Recruiting | thrombolytic drugs | Alteplase | Phase 4 | 400 | IIT | observational | 2020 | 2020/7/11 | single center |
| ChiCTR | ChiCTR-ORC-17012596 | 尿激酶溶栓治疗急性脑梗死 | Recruiting | thrombolytic drugs | Urokinase | Not Applicable | 1000 | IIT | observational | 2017 | 2017/9/6 | single center |
| ChiCTR | ChiCTR-TRC-13003024 | 不同剂量阿替普酶用于70岁及以上中国人群急性缺血性脑卒中静脉溶栓治疗有效性和安全性的一项前瞻性、多中心、随机、开放、对照临床研究 | Recruiting | thrombolytic drugs | Alteplase | Not Applicable | 500 | IIT | Interventional | 2013 | 2013/1/23 | single center |
| CDE | CTR20130997 | 三氟柳胶囊治疗和预防脑梗死的安全性和有效性 | Recruiting | antiplatelet drugs | Triflusal | Not Applicable | 240 | IST | Interventional | 2014 | 2014/11/19 | multiple center |
| CDE | CTR20131120 | 吡拉格雷钠注射液单次给药耐受性及初步PK-PD研究 | Completed | antiplatelet drugs | Pirragore sodium injection | Not Applicable | 62 | IST | Interventional | 2014 | 2014/7/7 | single center |
| CDE | CTR20131876 | 阿司匹林肠溶片生物等效性试验 | withdrawn | antiplatelet drugs | Aspirin | Not Applicable | 8 | IST | Interventional | 2016 | 2016/2/25 | single center |
| CDE | CTR20150719 | 三氟柳胶囊治疗和预防脑梗死的有效性和安全性研究 | Not yet recruiting | antiplatelet drugs | Triflusal | Phase 3 | 240 | IST | Interventional | 2015 | 2015/11/4 | multiple center |
| CDE | CTR20160214 | 健康志愿者中给药容积改变的耐受性及药代动力学研究 | Completed | antiplatelet drugs | Pirragore sodium injection | Phase 1 | 16 | IST | Interventional | 2016 | 2016/4/12 | single center |
| CDE | CTR20160215 | 单次给药药动学及24h连续给药的耐受性及药动学研究 | Completed | antiplatelet drugs | Pirragore sodium injection | Phase 1 | 16 | IST | Interventional | 2016 | 2016/4/12 | single center |
| CDE | CTR20180545 | 治疗急性缺血性脑卒中患者的有效性和安全性 | Recruiting | antiplatelet drugs | Pirragore sodium injection | Phase 2 | 400 | IST | Interventional | 2018 | 2018/5/11 | multiple center |
| CDE | CTR20180447 | 铭复乐治疗急性缺血性脑卒中（发病<3h）II期剂量探索研究 | Completed | thrombolytic drugs | rhTNK-tPA | Phase 2 | 240 | IST | Interventional | 2018 | 2018/5/10 | multiple center |
| CDE | CTR20190813 | 国人健康受试者单次静脉注射铭复乐的Ⅰ期临床试验 | Not yet recruiting | thrombolytic drugs | rhTNK-tPA | Phase 1 | 20 | IST | Interventional | 2019 | 2019/8/15 | single center |
| CDE | CTR20201936 | 注射用替奈普酶治疗超急性期（发病<4.5h）缺血性脑卒中的多中心、区组随机、开放性、终点盲法、阳性药物平行对照II临床试验（3TStroke-II） | Recruiting | thrombolytic drugs | Tenecteplase | Phase 2 | 225 | IST | Interventional | 2020 | 2020/9/29 | multiple center |
| Clinical trials. gov | NCT01923818 | Treatment of Rivaroxaban Versus Aspirin for Non-disabling Cerebrovascular Events | Unknown status | anticoagulant drugs | rivaroxaban\|Aspirin\|placebo | Phase 2\|Phase 3 | 3700 | IIT | Interventional | 2013 | 2013/9/1 | single center |
| Clinical trials. gov | NCT01924325 | Apixaban Versus Dual-antiplatelet Therapy (Clopidogrel and Aspirin) in Acute Non-disabling Cerebrovascular Events | Unknown status | anticoagulant drugs | Apixaban\|Clopidogrel\|Aspirin\|placebo | Phase 2\|Phase 3 | 10000 | IIT | Interventional | 2014 | 2014/1/1 | single center |
| Clinical trials. gov | NCT02221102 | Edoxaban for TIA and Acute Minor Stroke | Unknown status | anticoagulant drugs | Aspirin\|edoxaban\|placebo | Phase 2\|Phase 3 | 3700 | IIT | Interventional | 2013 | 2013/12/1 | single center |
| Clinical trials. gov | NCT03552354 | Argatroban Combined With Antiplatelet Versus Antiplatelet for Acute Ischemic Stroke | Completed | anticoagulant drugs | Argatroban plus dual antiplatelet | Phase 4 | 120 | IIT | Interventional | 2017 | 2017/10/25 | single center |
| Clinical trials. gov | NCT03740958 | Argatroban Plus R-tPA for Acute Ischemic Stroke | Recruiting | anticoagulant drugs | rt-PA\|Argatroban | Phase 4 | 808 | IIT | Interventional | 2018 | 2018/12/21 | single center |
| Clinical trials. gov | NCT03749057 | Early Rivaroxaban for Acute Ischemic Stroke or TIA Patients With Atrial Fibrillation | Recruiting | anticoagulant drugs | Rivaroxaban | Phase 4 | 1000 | IIT | Interventional | 2018 | 2018/11/20 | single center |
| Clinical trials. gov | NCT03772457 | Predictive Value of Infarction Volume on Hemorrhagic Transformation in Ischemic Stroke/TIA With Non-valve Atrial Fibrillation(NVAF) Patients Using Rivaroxaban | Recruiting | anticoagulant drugs | Rivaroxaban | unavailable | 400 | IST | Observational | 2019 | 2019/1/18 | single center |
| Clinical trials. gov | NCT04275180 | Clinical Study of Argatroban in the Treatment of Acute Progressive Ischemic Stroke | Recruiting | anticoagulant drugs | Argatroban | Phase 4 | 628 | IIT | Interventional | 2020 | 2020/3/21 | multiple center |
| Clinical trials. gov | NCT00979589 | Clopidogrel in High-risk Patients With Acute Non-disabling Cerebrovascular Events | Completed | antiplatelet drugs | Clopidogrel\|Placebo of clopidogrel and Asprin | Phase 3 | 5100 | IIT | Interventional | 2009 | 2009/12/1 | single center |
| Clinical trials. gov | NCT02506140 | Platelet Reactivity in Acute Non-disabling Cerebrovascular Events | Completed | antiplatelet drugs | Ticagrelor and Acetylsalicylic acid\|Clopidogrel and Acetylsalicylic acid | Phase 2\|Phase 3 | 675 | IIT | Interventional | 2015 | 2015/8/1 | multiple center |
| Clinical trials. gov | NCT02869009 | Antiplatelet Therapy in Acute Mild-Moderate Ischemic Stroke | Recruiting | antiplatelet drugs | clopidogrel\|Aspirin | Early Phase 1 | 3000 | IIT | Interventional | 2016 | 2016/11/1 | single center |
| Clinical trials. gov | NCT03357133 | Tirofiban for Patients Treated With Alteplase | Terminated | antiplatelet drugs | Tirofiban\|Alteplase | Phase 2\|Phase 3 | 30 | IIT | Interventional | 2017 | 2017/12/16 | multiple center |
| Clinical trials. gov | NCT03661411 | Antiplatelet vs R-tPA for Acute Mild Ischemic Stroke | Recruiting | antiplatelet drugs | Aspirin\|Clopidogrel 75mg\|Alteplase | Phase 4 | 760 | IIT | Interventional | 2018 | 2018/10/17 | single center |
| Clinical trials. gov | NCT03844594 | Eptifibatide in Endovascular Treatment of Acute Ischemic Stroke (EPOCH) | Unknown status | antiplatelet drugs | Eptifibatide Injection | Phase 3 | 220 | IIT | Interventional | 2019 | 2019/4/6 | single center |
| Clinical trials. gov | NCT03871517 | INdobufen Versus aSpirin in acUte Ischemic stRokE,INSURE | Not yet recruiting | antiplatelet drugs | Indobufen\|Aspirin | Phase 4 | 5390 | IIT | Interventional | 2019 | 2019/5/1 | single center |
| Clinical trials. gov | NCT04078737 | Clopidogrel With Aspirin in High-risk Patients With Acute Non-disabling Cerebrovascular Events II | Recruiting | antiplatelet drugs | Ticagrelor and Aspirin\|Clopidogrel and Aspirin | Phase 3 | 6396 | IIT | Interventional | 2019 | 2019/9/23 | multiple center |
| Clinical trials. gov | NCT04088513 | Safety and Efficacy of Aspirin in Stroke Patients With Glucose-6-phosphate Dehydrogenase Deficiency (SAST) | Recruiting | antiplatelet drugs | Aspirin\|Clopidogrel | Phase 4 | 440 | IIT | Interventional | 2020 | 2020/1/22 | multiple center |
| Clinical trials. gov | NCT04491695 | Tirofiban for the Prevention of Neurological Deterioration in Acute Ischemic Stroke | Recruiting | antiplatelet drugs | Tirofiban Hydrochloride\|Oral antiplatelet | Phase 2\|Phase 3 | 420 | IIT | Interventional | 2020 | 2020/9/12 | multiple center |
| Clinical trials. gov | NCT04504864 | Low-dose Aspirin Therapy in Patients With Ischemic Stroke and Microbleeds | Not yet recruiting | antiplatelet drugs | low-dose aspirin\|conventional-does aspirin | Phase 4 | 400 | IIT | Interventional | 2020 | 2020/8/1 | multiple center |
| Clinical trials. gov | NCT04624295 | Early Antiplatelet Therapy After Hemorrhagic Infarction in Acute Ischemic Stroke Treated With Intravenous Thrombolysis (HITs) | Recruiting | antiplatelet drugs | Early aspirin Therapy\|Non-Early aspirin Therapy | Phase 4 | 290 | IIT | Interventional | 2020 | 2020/11/1 | single center |
| Clinical trials. gov | NCT04851457 | One Pass Tirofiban In Management of Ischemic Stroke Thrombectomy In China | Not yet recruiting | antiplatelet drugs | Intravenous tirofiban combination therapy | Phase 2\|Phase 3 | 200 | IIT | Interventional | 2021 | 2021/5/1 | single center |
| Clinical trials. gov | NCT04952311 | Efficacy Study of Clopidogrel in High-risk Population With Acute Non-disabling Cerebrovascular Events Ⅱ | Recruiting | antiplatelet drugs | Tagrelor combined with aspirin\|Clopidogrel combined with aspirin | Phase 3 | 10878 | IIT | Interventional | 2019 | 2019/6/1 | single center |
| Clinical trials. gov | NCT04962451 | Comparison of the Efficacy of Ticagrelor Combined With ASA to ASA Alone in Patients With Stroke | Completed | antiplatelet drugs | ticagrelor + ASA\|Placebo+ASA | Phase 4 | 13000 | IST | Interventional | 2017 | 2017/9/1 | single center |
| Clinical trials. gov | NCT02854592 | Intravenous Thrombolysis Registry for Chinese Ischemic Stroke Within 4.5 h Onset | Completed | thrombolytic drugs | rtPA\|urokinase | unavailable | 4000 | IIT | Observational | 2017 | 2017/4/1 | single center |
| Clinical trials. gov | NCT02930837 | Safety and Efficacy of Alteplase When Administered in Chinese Patients With Acute Ischemic Hemispheric Stroke Where Thrombolysis is Initiated Between 3 and 4.5 Hours After Stroke Onset | Completed | thrombolytic drugs | alteplase | Phase 3 | 120 | IST | Interventional | 2016 | 2016/11/15 | multiple center |
| Clinical trials. gov | NCT03541668 | Study of rhPro-UK in Patients With Acute Ischaemic Stroke in 4.5 Hours After Stroke Onset(PROST) | Completed | thrombolytic drugs | Recombinant human urokinase\|Alteplase | Phase 3 | 674 | IST | Interventional | 2018 | 2018/5/18 | multiple center |
| Clinical trials. gov | NCT03578822 | Thrombolysis With rhPro-UK in 4.5-6 Hours After Acute Ischemic Stroke in a Double-blinded,Controlled Trial | Completed | thrombolytic drugs | Recombinant human urokinase\|Aspirin\|rhPro-UK simulation agent\|Aspirin simulation agent | Phase 3 | 149 | IST | Interventional | 2018 | 2018/8/10 | multiple center |
| Clinical trials. gov | NCT03733223 | Relationships Among FVIII, t-PA/PAI-1, and MMP-9 Levels and Intracranial Hemorrhage Complications After Thrombolysis With Alteplase in Patients With Acute Ischemic Stroke: Protocol for a Multicenter Retrospective Study | Unknown status | thrombolytic drugs | Ateptidase | unavailable | 350 | IIT | Observational | 2018 | 2018/10/12 | single center |
| Clinical trials. gov | NCT03997292 | Intravenous Thrombolysis Registry for Acute Ischemic Stroke in China | Withdrawn | thrombolytic drugs | Alteplase\|Urokinase | unavailable | 0 | IIT | Observational | 2018 | 2018/3/1 | single center |
| Clinical trials. gov | NCT04028518 | A PhaseⅡ of Injection for Recombinant Human Tissue Plasminogen Kinase Derivative in Treatment of Acute Ischemic Stroke. | Unknown status | thrombolytic drugs | r-PA\|Alteplase for Injection | Phase 2 | 180 | IST | Interventional | 2019 | 2019/7/20 | single center |
| Clinical trials. gov | NCT04086147 | CHinese Acute Tissue-Based Imaging Selection for Lysis In Stroke -Tenecteplase | Recruiting | thrombolytic drugs | Low dose tenecteplase\|High dose tenecteplase | Phase 2 | 86 | IIT | Interventional | 2019 | 2019/12/9 | single center |
| Clinical trials. gov | NCT04201964 | Improving Neuroprotective Strategy for Ischemic Stroke With Poor Recanalization After Thrombectomy by Intra-arterial TNK (INSIST-TNK) | Recruiting | thrombolytic drugs | Intra-arterial administration of tenecteplase | Not Applicable | 30 | IIT | Interventional | 2019 | 2019/12/15 | single center |
| Clinical trials. gov | NCT04202458 | Boosting REcanalization of Thrombectomy for Ischemic Stroke by Intra-arterial TNK (BRETIS-TNK) | Recruiting | thrombolytic drugs | intra-arterial tenecteplase administration | Not Applicable | 30 | IIT | Interventional | 2019 | 2019/12/15 | single center |
| Clinical trials. gov | NCT04290494 | Temporal Trends of Thrombolysis Treatment in Chinese Acute Ischemic Stroke (AIS) Patients From 2007-2017: Analysis of China National Stroke Registry (CNSR) I, II, and III | Completed | thrombolytic drugs | IV rtPA (intravenous recombinant plasminogen activator) | unavailable | 42188 | IST | Observational | 2020 | 2020/9/21 | single center |
| Clinical trials. gov | NCT04420351 | Thrombolysis of Urokinase for Minor Stroke | Not yet recruiting | thrombolytic drugs | Urokinase thrombolysis\|Aspirin;Clopidogrel | Phase 3 | 1002 | IIT | Interventional | 2020 | 2020/6/10 | multiple center |
| Clinical trials. gov | NCT04516993 | CHinese Acute Tissue-Based Imaging Selection for Lysis In Stroke -Tenecteplase II | Not yet recruiting | thrombolytic drugs | Tenecteplase\|nonthrombolysis drug | Phase 2 | 224 | IIT | Interventional | 2021 | 2021/1/1 | single center |
| Clinical trials. gov | NCT04588337 | INtravenous TNK for Acute isChemicsTroke in China | Not yet recruiting | thrombolytic drugs | rhTNK-tPA | unavailable | 1000 | IIT | Observational | 2021 | 2021/7/10 | single center |
| Clinical trials. gov | NCT04676659 | Tenecteplase Reperfusion Therapy in Acute Ischemic Cerebrovascular Events(TRACE) | Completed | thrombolytic drugs | TNK-tPA | Phase 2 | 240 | IST | Interventional | 2018 | 2018/5/12 | multiple center |
| Clinical trials. gov | NCT04733742 | Endovascular Treatment With Versus Without Intravenous Tenecteplase in Stroke | Not yet recruiting | thrombolytic drugs | Tenecteplase\|Other: Endovascular treatment | Phase 2\|Phase 3 | 542 | IIT | Interventional | 2021 | 2021/12/1 | multiple center |
| Clinical trials. gov | NCT04797013 | Tenecteplase Reperfusion Therapy in Acute Ischemic Cerebrovascular Events-Ⅱ | Recruiting | thrombolytic drugs | rt-PA\|rhTNK-tPA | Phase 3 | 1430 | IST | Interventional | 2021 | 2021/3/31 | multiple center |
| Clinical trials. gov | NCT04879615 | 24 Hours Treatment With Alteplase in Patients With Ischemic Stroke | Recruiting | thrombolytic drugs | Alteplase | Phase 3 | 372 | IIT | Interventional | 2021 | 2021/6/21 | single center |
| Clinical trials. gov | NCT04915729 | A Study in Chinese Patients to Compare How Tenecteplase and Alteplase Given After a Stroke Improve Recovering of Physical Activity | Recruiting | thrombolytic drugs | Tenecteplase\|alteplase | Phase 3 | 800 | IST | Interventional | 2021 | 2021/6/22 | multiple center |
