# Supplementary material for: Evolution of ischemic stroke drug clinical trials in mainland China from 2005 to 2021
Source: CNS Neurosci Ther. 2022 Jun 1;28(8):1229–39. doi: 10.1111/cns.13867 (PMC9253749; doi:10.1111/cns.13867)
Supplement: Supplementary file 7 — Table S4 [file CNS-28-1229-s004.docx]

**Table S4. Clinical trials including combinations of different types of drugs.**

| **Database** | **NCT Number** | **Title** | **Drug type** | **Interventions** | **Study Type** | **Phases** | **Status** | **Enrollment** | **IIT/IST** | **Start year** | **Start Date** | **Single center or multiple center** |
| --- | --- | --- | --- | --- | --- | --- | --- | --- | --- | --- | --- | --- |
| Clinical Trials.gov | NCT03552354 | Argatroban Combined With Antiplatelet Versus Antiplatelet for Acute Ischemic Stroke | Antithrombotic therapy | Argatroban plus dual antiplatelet | Interventional | Phase 4 | Completed | 120 | IIT | 2017 | 2017/10/25 | single center |
| Clinical Trials.gov | NCT03740958 | Argatroban Plus R-tPA for Acute Ischemic Stroke | Antithrombotic therapy | rt-PA\| Argatroban | Interventional | Phase 4 | Recruiting | 808 | IIT | 2018 | 2018/12/21 | single center |
| Clinical Trials.gov | NCT01924325 | Apixaban Versus Dual-antiplatelet Therapy (Clopidogrel and Aspirin) in Acute Non-disabling Cerebrovascular Events | Antithrombotic therapy | Apixaban\| Clopidogrel\| Aspirin\| placebo | Interventional | Phase 2\|Phase 3 | Unknown status | 10000 | IIT | 2014 | 2014/1/1 | single center |
| Clinical Trials.gov | NCT01923818 | Treatment of Rivaroxaban Versus Aspirin for Non-disabling Cerebrovascular Events | Antithrombotic therapy | rivaroxaban\| Aspirin\| placebo | Interventional | Phase 2\|Phase 3 | Unknown status | 3700 | IIT | 2013 | 2013/9/1 | single center |
| Clinical Trials.gov | NCT04088513 | Safety and Efficacy of Aspirin in Stroke Patients With Glucose-6-phosphate Dehydrogenase Deficiency (SAST) | Antithrombotic therapy | Aspirin\| Clopidogrel | Interventional | Phase 4 | Recruiting | 440 | IIT | 2020 | 2020/1/22 | multiple center |
| Clinical Trials.gov | NCT02869009 | Antiplatelet Therapy in Acute Mild-Moderate Ischemic Stroke | Antithrombotic therapy | clopidogrel\| Aspirin | Interventional | Early Phase 1 | Recruiting | 3000 | IIT | 2016 | 2016/11/1 | single center |
| Clinical Trials.gov | NCT00979589 | Clopidogrel in High-risk Patients With Acute Non-disabling Cerebrovascular Events | Antithrombotic therapy | Clopidogrel\| Placebo of clopidogrel and Asprin | Interventional | Phase 3 | Completed | 5100 | IIT | 2009 | 2009/12/1 | single center |
| Clinical Trials.gov | NCT03661411 | Antiplatelet vs R-tPA for Acute Mild Ischemic Stroke | Antithrombotic therapy | Aspirin\| Clopidogrel 75mg\| Alteplase | Interventional | Phase 4 | Recruiting | 760 | IIT | 2018 | 2018/10/17 | single center |
| Clinical Trials.gov | NCT04962451 | Comparison of the Efficacy of Ticagrelor Combined With ASA to ASA Alone in Patients With Stroke | Antithrombotic therapy | ticagrelor + ASA\| Placebo+ASA | Interventional | Phase 4 | Completed | 13000 | IST | 2017 | 2017/9/1 | single center |
| Clinical Trials.gov | NCT04078737 | Clopidogrel With Aspirin in High-risk Patients With Acute Non-disabling Cerebrovascular Events II | Antithrombotic therapy | Ticagrelor and Aspirin\| Clopidogrel and Aspirin | Interventional | Phase 3 | Recruiting | 6396 | IIT | 2019 | 2019/9/23 | multiple center |
| Clinical Trials.gov | NCT02506140 | Platelet Reactivity in Acute Non-disabling Cerebrovascular Events | Antithrombotic therapy | Ticagrelor and Acetylsalicylic acid\| Clopidogrel and Acetylsalicylic acid | Interventional | Phase 2\|Phase 3 | Completed | 675 | IIT | 2015 | 2015/8/1 | multiple center |
| Clinical Trials.gov | NCT04952311 | Efficacy Study of Clopidogrel in High-risk Population With Acute Non-disabling Cerebrovascular Events Ⅱ | Antithrombotic therapy | Tagrelor combined with aspirin\| Clopidogrel combined with aspirin | Interventional | Phase 3 | Recruiting | 10878 | IIT | 2019 | 2019/6/1 | single center |
| Clinical Trials.gov | NCT04491695 | Tirofiban for the Prevention of Neurological Deterioration in Acute Ischemic Stroke | Antithrombotic therapy | Tirofiban Hydrochloride\| Oral antiplatelet | Interventional | Phase 2\|Phase 3 | Recruiting | 420 | IIT | 2020 | 2020/9/12 | multiple center |
| Clinical Trials.gov | NCT03357133 | Tirofiban for Patients Treated With Alteplase | Antithrombotic therapy | Tirofiban\| Alteplase | Interventional | Phase 2\|Phase 3 | Terminated | 30 | IIT | 2017 | 2017/12/16 | multiple center |
| Clinical Trials.gov | NCT03871517 | INdobufen Versus aSpirin in acUte Ischemic stRokE,INSURE | Antithrombotic therapy | Indobufen\| Aspirin | Interventional | Phase 4 | Not yet recruiting | 5390 | IIT | 2019 | 2019/5/1 | single center |
| Clinical Trials.gov | NCT04028518 | A PhaseⅡ of Injection for Recombinant Human Tissue Plasminogen Kinase Derivative in Treatment of Acute Ischemic Stroke. | Antithrombotic therapy | r-PA\| Alteplase for Injection | Interventional | Phase 2 | Unknown status | 180 | IST | 2019 | 2019/7/20 | single center |
| Clinical Trials.gov | NCT03997292 | Intravenous Thrombolysis Registry for Acute Ischemic Stroke in China | Antithrombotic therapy | Alteplase\| Urokinase | Observational | NA | Withdrawn | 0 | IIT | 2018 | 2018/3/1 | single center |
| Clinical Trials.gov | NCT02854592 | Intravenous Thrombolysis Registry for Chinese Ischemic Stroke Within 4.5 h Onset | Antithrombotic therapy | rtPA\| urokinase | Observational | NA | Completed | 4000 | IIT | 2017 | 2017/4/1 | single center |
| Clinical Trials.gov | NCT04420351 | Thrombolysis of Urokinase for Minor Stroke | Antithrombotic therapy | Urokinase thrombolysis\| Aspirin;Clopidogrel | Interventional | Phase 3 | Not yet recruiting | 1002 | IIT | 2020 | 2020/6/10 | multiple center |
| Clinical Trials.gov | NCT04797013 | Tenecteplase Reperfusion Therapy in Acute Ischemic Cerebrovascular Events-Ⅱ | Antithrombotic therapy | rt-PA\| rhTNK-tPA | Interventional | Phase 3 | Recruiting | 1430 | IST | 2021 | 2021/3/31 | multiple center |
| Clinical Trials.gov | NCT03578822 | Thrombolysis With rhPro-UK in 4.5-6 Hours After Acute Ischemic Stroke in a Double-blinded,Controlled Trial | Antithrombotic therapy | Recombinant human urokinase\| Aspirin\| rhPro-UK simulation agent\| Aspirin simulation agent | Interventional | Phase 3 | Completed | 149 | IST | 2018 | 2018/8/10 | multiple center |
| Clinical Trials.gov | NCT03541668 | Study of rhPro-UK in Patients With Acute Ischaemic Stroke in 4.5 Hours After Stroke Onset(PROST) | Antithrombotic therapy | Recombinant human urokinase\| Alteplase | Interventional | Phase 3 | Completed | 674 | IST | 2018 | 2018/5/18 | multiple center |
| Clinical Trials.gov | NCT04516993 | CHinese Acute Tissue-Based Imaging Selection for Lysis In Stroke -Tenecteplase II | Antithrombotic therapy | Tenecteplase\| nonthrombolysis drug | Interventional | Phase 2 | Not yet recruiting | 224 | IIT | 2021 | 2021/1/1 | single center |
| Clinical Trials.gov | NCT04733742 | Endovascular Treatment With Versus Without Intravenous Tenecteplase in Stroke | Antithrombotic therapy | Tenecteplase\| Endovascular treatment | Interventional | Phase 2\|Phase 3 | Not yet recruiting | 542 | IIT | 2021 | 2021/12/1 | multiple center |
| Clinical Trials.gov | NCT04915729 | A Study in Chinese Patients to Compare How Tenecteplase and Alteplase Given After a Stroke Improve Recovering of Physical Activity | Antithrombotic therapy | tenecteplase\| alteplase | Interventional | Phase 3 | Recruiting | 800 | IST | 2021 | 2021/6/22 | multiple center |
| Clinical Trials.gov | NCT02149875 | Dl-3-n-butylphthalide and Cerebrolysin Treatment in Acute Ischemic Stroke | Cerebral protection | Dl-3-n-butylphthalide\| Cerebrolysin\| Placebo | Interventional | Phase 1\|Phase 2 | Completed | 84 | IIT | 2010 | 2010/1/1 | single center |
| Clinical Trials.gov | NCT04984577 | Study of Compound Edaravone Injection for Treatment of Acute Ischemic Stroke | Cerebral protection | Compound Edaravone Injection-Low dose\| Compound Edaravone Injection-High dose\| Edaravone Injection\| Placebo injection | Interventional | Phase 2 | Not yet recruiting | 240 | IST | 2021 | 2021/9/1 | single center |
| Clinical Trials.gov | NCT02430350 | Study of Compound Edaravone Injection for Treatment of Acute Ischemic Stroke | Cerebral protection | Compound Edaravone Injection\| Edaravone Injection | Interventional | Phase 3 | Completed | 1200 | IST | 2015 | 2015/5/1 | multiple center |
| Clinical Trials.gov | NCT01929096 | Compound Edaravone Injection for Acute Ischemic Stroke | Cerebral protection | Compound Edaravone Injection\| Edaravone Injection | Interventional | Phase 2 | Completed | 400 | IST | 2013 | 2013/8/1 | multiple center |
| Clinical Trials.gov | NCT03394950 | Butyphthalide in Combination With Recombinant Tissue Plasminogen Activator for Acute Ischemic Stroke | Cerebral protection + Antithrombotic therapy | Butyphthalide combined with rtPA\| rtPA | Interventional | Phase 4 | Completed | 120 | IIT | 2018 | 2018/5/25 | single center |
| Clinical Trials.gov | NCT00724724 | The Effectiveness and Safety of Butylphthalide Soft Capsules in Secondary Prevention of Ischemic Stroke Trial | Cerebral protection + Antithrombotic therapy | Butylphthalide Soft Capsules\| Aspirin | Interventional | Phase 4 | Unknown status | 1000 | IIT | 2008 | 2008/8/1 | multiple center |
| Clinical Trials.gov | NCT01831011 | Mildronate for Acute Ischemic Stroke | Cerebral protection + Antithrombotic therapy | mildronate injection\| cinepazide maleate injection\| aspirin | Interventional | Phase 2 | Completed | 227 | IIT | 2008 | 2008/7/1 | single center |
| Clinical Trials.gov | NCT01800357 | Efficacy and Safety of Mildronate for Acute Ischemic Stroke | Cerebral protection + Antithrombotic therapy | infusion of mildronate\| placebo\| aspirin | Interventional | Phase 2 | Unknown status | 240 | IIT | 2013 | 2013/1/1 | single center |
| Clinical Trials.gov | NCT03753555 | The Effect of InTensive Statin in Ischemic Stroke With inTracranial Atherosclerotic Plaques | Lipid-lowering therapy | Atorvastatin Calcium\| Probucol | Interventional | Phase 4 | Recruiting | 100 | IIT | 2018 | 2018/12/1 | single center |
| ChiCTR | ChiCTR2100045812 | 阿加曲班联合双重抗血小板治疗超溶栓时间窗分水岭脑梗死的疗效和安全性评价：一个多中心、前瞻性、开放标签的随机对照临床试验 | Antithrombotic therapy | Agaltreban \| dual antiplatelet | Interventional | Phase 4 | Recruiting | 240 | IIT | 2021 | 2021/4/25 | single center |
| ChiCTR | ChiCTR-OPN-16007931 | 肝素、双抗（波立维及阿司匹林）及单抗（阿司匹林）治疗急性期脑梗死及TIA的临床研究 | Antithrombotic therapy | Heparin \| clopidogrel and aspirin \| aspirin | Interventional | Phase 4 | Recruiting | 588 | IIT | 2016 | 2016/2/16 | single center |
| ChiCTR | ChiCTR2000030824 | 阿司匹林、氯吡格雷联合丁苯酞对中老年急性缺血性脑卒中患者精神、神经和行为功能的影响 | Antithrombotic therapy | Aspirin and clopidogrel combined with butylphthalein | Interventional | Phase 0 | Recruiting | 200 | IIT | 2020 | 2020/3/15 | single center |
| ChiCTR | ChiCTR-IPR-15006826 | 双抗和降纤治疗急性大动脉粥样硬化性脑梗死的随机、平行对照、多中心临床比较研究 | Antithrombotic therapy | Dual antiplatelet \| defibration therapy | Interventional | Phase 4 | Recruiting | 400 | IIT | 2015 | 2015/7/28 | single center |
| ChiCTR | ChiCTR2000029556 | 低剂量rt-PA联合替罗非班治疗DWI-Flair不匹配的发病时间不明的急性非心源性脑梗死的临床安全及疗效 | Antithrombotic therapy | Low dose rt-PA combined with tirofiban | Interventional | Phase 0 | Not yet recruiting | 80 | IIT | 2020 | 2020/2/4 | single center |
| ChiCTR | ChiCTR2000029023 | 阿加曲班联合阿替普酶治疗急性脑梗死的有效性和安全性研究 | Antithrombotic therapy | Agaltreban combined with alteplase | Interventional | Phase 0 | Recruiting | 100 | IIT | 2020 | 2020/1/11 | single center |
| ChiCTR | ChiCTR1800014666 | 低剂量rt-PA联合替罗非班治疗 非心源性急性脑梗死的临床疗效研究 | Antithrombotic therapy | Low dose rt-PA combined with tirofiban | Interventional | Phase 2 | Recruiting | 100 | IIT | 2018 | 2018/1/28 | single center |
| ChiCTR | ChiCTR2000029401 | L-精氨酸及ADMA在急性脑梗死患者早期神经功能恶化和早期复发中的作用 | Cerebral protection | L-arginine \|ADMA | Interventional | Phase 0 | Recruiting | 240 | IIT | 2020 | 2020/1/29 | single center |
| ChiCTR | ChiCTR-IPR-17010545 | 大面积脑梗死患者渗透性药物治疗研究：甘露醇与高渗盐对比 | Dehydrant agents | Mannitol and hypertonic salts | Interventional | Phase 4 | Recruiting | 80 | IIT | 2017 | 2017/2/3 | multiple center |
| ChiCTR | ChiCTR2000035887 | 利拉鲁肽联合利格列汀改善急性缺血性脑卒中合并高血糖患者神经功能的随机对照临床研究 | Hypoglycemic therapy | Liraglutide \| ligagliptin | Interventional | Phase 4 | Not yet recruiting | 680 | IIT | 2020 | 2020/8/19 | single center |
| ChiCTR | ChiCTR2000040461 | 普罗布考联合阿托伐他汀治疗急性缺血性脑卒中效果评价——一个随机同期对照试验 | Lipid-lowering therapy | Probucol \| atorvastatin | Interventional | Phase 0 | Recruiting | 80 | IIT | 2020 | 2020/11/28 | single center |
| ChiCTR | ChiCTR2100044882 | 再灌注联合神经保护治疗后循环脑梗死的临床研究 | Reperfusion + Cerebral protection | Reperfusion combined with neuroprotective therapy | Interventional | Phase 4 | Recruiting | 60 | IIT | 2021 | 2021/3/31 | single center |
| ChiCTR | ChiCTR2000028795 | 围术期联合应用右美托咪定和阿片类药物对急性缺血性脑卒中患者机械取栓的影响 | Sedative drugs | Dexmedetomidine and opioids | Interventional | Phase 4 | Not yet recruiting | 150 | IIT | 2020 | 2020/1/4 | single center |
| ChiCTR | ChiCTR1800020020 | 右美托咪定复合乌司他丁对急性缺血性脑卒中行介入治疗手术患者早期神经功能改善及预后的影响：前瞻、双盲、随机对照临床试验 | Sedative drugs | Dexmedetomidine combined with ulinastatin | Interventional | NA | Not yet recruiting | 150 | IIT | 2018 | 2018/12/11 | single center |
| ChiCTR | ChiCTR2000038727 | 冬眠合剂（氯丙嗪+异丙嗪）对急性缺血性脑卒中神经保护作用：临床前瞻性随机对照研究 | Sedative drugs | Hibernation mixture (chlorpromazine + promethazine) | Interventional | Phase 4 | Not yet recruiting | 166 | IIT | 2020 | 2020/9/29 | single center |
